# Differentiation but not ALS mutations in FUS rewires motor neuron metabolism

**DOI:** 10.1038/s41467-019-12099-4

**Published:** 2019-09-12

**Authors:** Tijs Vandoorne, Koen Veys, Wenting Guo, Adria Sicart, Katlijn Vints, Ann Swijsen, Matthieu Moisse, Guy Eelen, Natalia V. Gounko, Laura Fumagalli, Raheem Fazal, Christine Germeys, Annelies Quaegebeur, Sarah-Maria Fendt, Peter Carmeliet, Catherine Verfaillie, Philip Van Damme, Bart Ghesquière, Katrien De Bock, Ludo Van Den Bosch

**Affiliations:** 1 Department of Neurosciences, Experimental Neurology, and Leuven Brain Institute, KU Leuven – University of Leuven, Leuven, Belgium; 2 VIB, Center for Brain & Disease Research, Laboratory of Neurobiology, Leuven, Belgium; 3 Department of Oncology, Laboratory of Angiogenesis and Vascular Metabolism, KU Leuven – University of Leuven, Leuven, Belgium; 4 VIB, Center for Cancer Biology, Laboratory of Angiogenesis and Vascular Metabolism, Leuven, Belgium; 5 Department of Development and Regeneration, Stem Cell Institute, KU Leuven – University of Leuven, Leuven, Belgium; 6 VIB, Center for Brain & Disease Research, Electron Microscopy Platform and VIB Bioimaging core facility, Leuven, Belgium; 7 Department of Neurosciences and Leuven Brain Institute, KU Leuven – University of Leuven, Leuven, Belgium; 8 Division of Neuropathology, The National Hospital for Neurology and Neurosurgery, University College London Hospitals NHS Foundation Trust, London, UK; 9 VIB, VIB-KU Leuven Center for Cancer Biology, Laboratory of Cellular Metabolism and Metabolic Regulation, Leuven, Belgium; 10 Department of Oncology, Laboratory of Cellular Metabolism and Metabolic Regulation, KU Leuven and Leuven Cancer Institute (LKI), Leuven, Belgium; 11 Department of Neurology, University Hospitals Leuven, Leuven, Belgium; 12 Department of Oncology, Metabolomics Core Facility, KU Leuven – University of Leuven, Leuven, Belgium; 13 VIB, Department of Oncology, Metabolomics Core Facility, Leuven, Belgium; 14 ETH Zürich, Department of Health Sciences and Technology, Laboratory of Exercise and Health, Zürich, Switzerland

**Keywords:** Cellular neuroscience, Molecular neuroscience, Neurodegeneration

## Abstract

Energy metabolism has been repeatedly linked to amyotrophic lateral sclerosis (ALS). Yet, motor neuron (MN) metabolism remains poorly studied and it is unknown if ALS MNs differ metabolically from healthy MNs. To address this question, we first performed a metabolic characterization of induced pluripotent stem cells (iPSCs) versus iPSC-derived MNs and subsequently compared MNs from ALS patients carrying FUS mutations to their CRISPR/Cas9-corrected counterparts. We discovered that human iPSCs undergo a lactate oxidation-fuelled prooxidative metabolic switch when they differentiate into functional MNs. Simultaneously, they rewire metabolic routes to import pyruvate into the TCA cycle in an energy substrate specific way. By comparing patient-derived MNs and their isogenic controls, we show that ALS-causing mutations in FUS did not affect glycolytic or mitochondrial energy metabolism of human MNs in vitro. These data show that metabolic dysfunction is not the underlying cause of the ALS-related phenotypes previously observed in these MNs.

## Abstract (web summary)

While energy metabolism has been repeatedly linked to ALS, motor neuron metabolism remains poorly studied. Here, authors show that human iPSCs rewire specific metabolic routes when they differentiate into functional motor neurons and that ALS-causing mutations in FUS do not affect energy metabolism.

## Introduction

Amyotrophic lateral sclerosis (ALS) is a fatal neurodegenerative disorder characterised by the selective degeneration of motor neurons (MNs) in the motor cortex, brainstem and spinal cord. MN deterioration leads to muscle weakness and results in death due to respiratory failure, usually within 3 to 5 years after diagnosis^1^. While 90% of ALS cases are sporadic, 10% are inherited. The most prevalent genetic causes of ALS are mutations in the ‘*superoxide dismutase 1’* (SOD1), ‘*fused in sarcoma’* (FUS), ‘*TAR DNA binding protein’* (TARDBP) gene, or a hexanucleotide repeat expansion in the ‘*chromosome 9 open reading frame 72’* (C9ORF72) gene^2^. Despite our increased understanding of the genetic factors contributing to ALS, the precise mechanisms underlying the selective MN degeneration in ALS remain enigmatic, and no effective treatments are available. As a consequence, more than 80,000 patients who are alive at present will succumb to the disease^3^.

A longstanding hypothesis states that bioenergetic failure in ALS MNs causes MN degeneration in ALS^4^. This hypothesis is based on several observations, which we recently reviewed^5^. Fast-fatigable MNs, which have the highest peak needs of ATP^6^, are initially targeted and are more severely affected in ALS compared with slow MNs^7^. Aberrant mitochondrial morphology and impaired mitochondrial function are observed in *postmortem* samples from ALS patients^8,9^, but are also early features in the central nervous system of various ALS rodent models^10–12^. In addition, glycogen stores are elevated in *postmortem* spinal cord of ALS patients, suggesting a reduced capacity to recruit and/or catabolize glucose^13^. Moreover, systemic metabolism correlates to disease course in ALS patients. For example, ALS patients suffering from diabetes show a delay in the onset of motor symptoms for up to 4 years^14^.

Recently, we and others found that ALS-causing FUS mutations affect the two most energy demanding biological processes in MNs, neuronal firing and axonal transport, using human-induced pluripotent stem cell (iPSC)-derived MNs^15–18^. FUS is a DNA/RNA-binding protein involved in both ALS and frontotemporal dementia (FTD). Compared with wild-type FUS, ALS-mutant FUS is mislocalized to the cytoplasm and has an increased interaction with enzymes involved in glucose metabolism^19^. In addition, FUS overexpression in flies, mice and in vitro impairs mitochondrial structure and bioenergetics^10,19–21^. Given the vulnerability of MNs to energetic stress^6^, this raises the question whether energy metabolism could drive the selective MN degeneration observed in ALS.

At present, most support for impaired energy metabolism in ALS comes from rodent models which overexpress disease-related proteins, or *postmortem* patient tissue. For this reason, a unifying view on how different metabolic pathways converge and whether metabolic alterations contribute to the disease aetiology in ALS is lacking. In fact, it is not known whether ALS MNs metabolically differ from healthy MNs. iPSC technology enables the validation and extension of hypotheses originating from rodent models in the context of human MNs. The generation of isogenic lines, in which the mutation is corrected, allows to infer causality from the disease-causing mutation on any finding and further extends the biological relevance of these models^22–24^. Using isogenic cell lines, we previously discovered that FUS point mutations cause defects in axonal transport and MN excitability^15^.

Taking advantage of these tools, we decided to unravel the metabolic profile of human iPSC-derived MNs and to investigate the effect of ALS-mutant FUS on MN metabolism. By combining CRISPR/Cas9-induced gene editing and state-of-the-art metabolic labelling techniques, we initially compared human iPSCs with iPSC-derived MNs and subsequently evaluated the effect of ALS mutations in FUS on MN metabolism. We show that iPSCs, as they differentiate into functional MNs, shift towards a more oxidative phenotype as they reduce glucose uptake and glycolytic flux, but elevate TCA cycle activity and respiratory potential. Interestingly, we observed that the increase in oxidative metabolism upon differentiation was mainly fuelled by a pronounced increase in lactate oxidation. Using this as a baseline, we show that two different ALS mutations in FUS do not affect energy metabolism in functional human iPSC-derived MNs. Our data exclude dysfunctional energy metabolism as the underlying cause of axonal transport defects or hypoexcitability observed in these MNs.

## Results

### Generation of functional MNs from human iPSCs

**Fig. 1:**
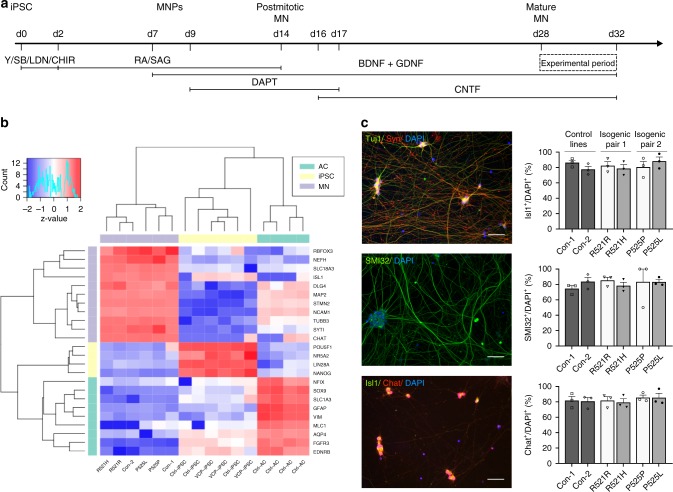
Characterisation of iPSC-derived MNs from ALS patients and controls. **a** Schematic overview of motor neuron differentiation from iPSCs with indication of timing when motor neuron experiments were performed. **b** Bulk RNA-sequencing analysis indicating enrichment of motor neuron-specific transcripts in human iPSC-derived motor neurons versus iPSCs and astrocytes. Raw data are deposited publicly, EGA (EGAS00001003785). **c** Representative staining of pan-neuronal markers (Tuj1 and Synapsin1) and staining and quantification of motor neuron markers (Isl1, ChAT and SMI-32) in motor neurons from controls (black bars; Con-1, Con-2), FUS-patients (dark grey bars; R521H, P525L) or isogenic controls from these patients (light grey bars; R521R, P525P). Scale bar, 50 μm. Motor neurons were between 28 and 32 days old for all experiments. Statistical analyses in panel **c** was performed by one-way ANOVA with post hoc two-tailed *t* tests between cell lines. In **c**, data are presented as mean ± s.e.m. from three individual experiments, with the individual data points shown. Source data are provided as a source data file. iPSC induced pluripotent stem cell, MNPs motor neuron progenitors, MN motor neuron, Y Y-27632, SB SB 431542, LDN LDN-193189, CHIR CHIR99021, RA retinoic acid, SAG smoothened agonist, DAPT a γ-secretase inhibitor, BDNF brain-derived neurotrophic factor, GDNF glial cell-derived neurotrophic factor, CNTF ciliary neurotrophic factor, AC astrocyte, Con-1 control cell line 1, Con-2 control cell line 2, R521H cell line derived from a patient carrying the R521H mutation in FUS, R521R CRISPR-Cas9-corrected R521H, P525L cell line derived from a patient carrying the P525L mutation in FUS, P525P CRISPR-Cas9-corrected P525L.

To investigate the energy metabolism of human MNs and iPSCs, we used six different iPSC lines. Two lines were generated from fibroblasts of ALS patients carrying a heterozygous mutation in the nuclear localisation signal of FUS: one patient carried a R521H mutation, whereas the other patient had a P525L mutation. In ALS, FUS is most commonly mutated at position 521, but the P525L mutation is notorious for causing aggressive juvenile-onset ALS^25,26^. To exclusively study the effect of the point mutation, we used isogenic control lines for both patients (R521R and P525P), which we generated previously^15,27^. Two additional lines were generated from healthy control subjects (Con-1 and Con-2) not carrying any ALS mutation (Supplementary Table 1). To differentiate iPSCs into MNs, we used a slightly modified version of the Maury protocol^15^ (Fig. 1a). Transcriptome profiling of our MNs showed enrichment of MN-specific genes versus iPSC and astrocyte markers, which were retrieved from a publicly available dataset (Fig. 1b)^28^. A high degree of transcriptional similarity was found between patient lines and isogenic controls, underscoring the value of using corrected patient lines to overcome heterogeneity (Supplementary Fig. 1). Approximately 80% of cells stained positive for the pan-neuronal markers Tuj1 and synapsin1 and for the MN-specific markers ChAT, Isl1 and SMI32 without significant differences between any of the lines (Fig. 1c). In addition, MNs were electrically active and fired both spontaneous and evoked action potentials, as shown using Con-1 (Supplementary Fig. 2). This is all in line with our previous observations^15^.

### Metabolic fate of lactate and glucose in human iPSCs and MNs

Metabolic characteristics of neurons have almost exclusively been studied in rodent cortical neurons^5^. We therefore first aimed to determine the metabolic properties of human MNs and performed a detailed metabolic characterisation of iPSC-derived MN metabolism compared with undifferentiated iPSCs. For this purpose, we used four different human control iPSC lines (Con-1, Con-2, R521R and P525P).

**Fig. 2:**
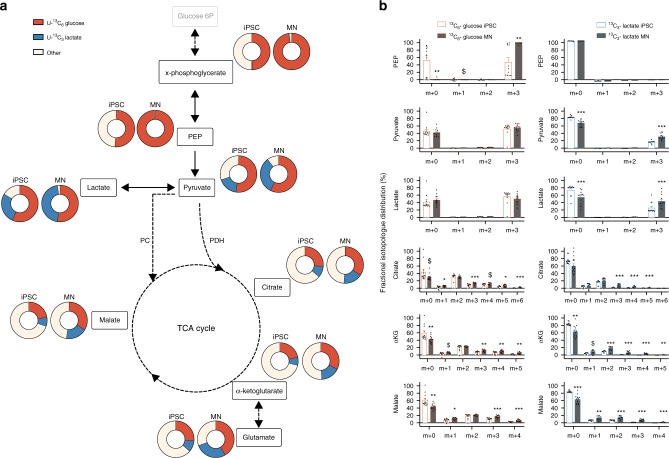
Metabolic fate of lactate and glucose in human iPSCs and MNs. **a** Fractional contribution of uniformly labelled ^13^C glucose (red) and uniformly labelled ^13^C lactate (blue) to metabolites from glycolysis and TCA cycle in human iPSCs and motor neurons. **b** Relative abundance of each isotopologue for specific metabolites. ^$^*p* < 0.10, *p < 0.05, **p < 0.01, ***p < 0.0001 versus iPSC. Statistical analyses were performed by two-tailed *t* tests to compare iPSCs and motor neurons. The data represent mean ± s.e.m. from four control cell lines from three independent experiments, with individual data points shown. Thin black arrows represent direct metabolic pathway connections; dashed arrows indicate that additional intermediate reactions are involved in the metabolic pathway. Source data are provided as a source data file. ^13^C_6_-glucose uniformly labelled glucose, ^13^C_3_-lactate uniformly labelled lactate, PEP phosphoenolpyruvate, PC pyruvate carboxylase, PDH pyruvate dehydrogenase, αKG alpha-ketoglutarate, TCA tricarboxylic acid

Under resting conditions, cortical neurons largely rely on glucose as energy substrate to meet their substantial energy demand^29^. As soon as they get activated, the utilisation of both glucose and lactate is increased^30^. To analyse the metabolic fate of glucose and lactate in iPSCs and MNs, we cultured the cells in medium containing either 5 mM uniformly labelled ^13^C-glucose (^13^C_6_-glucose) or 2 mM uniformly labelled ^13^C-lactate (^13^C_3_-lactate; Fig. 2). Incorporation of the ^13^C label into carbons of glycolytic and tricarboxylic acid (TCA) metabolites was subsequently analysed. Both cell types catabolized lactate, as indicated by the appearance of lactate-derived ^13^C-label into the total carbon pool of pyruvate and TCA metabolites (Fig. 2a, indicated in blue). Nonetheless, the fractional contribution of glucose (Fig. 2a, shown in red), showing the relatively contribution of the glucose-derived ^13^C-label in the total carbon pool of a metabolite, was significantly higher than the contribution of lactate for each metabolite in both iPSCs and MNs (*p* < 0.05). Independent of the metabolite, we found that the combined ^13^C-lactate and ^13^C-glucose enrichment was on average 57 ± 12% higher in MNs than in iPSCs (*p* < 0.0001), underscoring their dependence on glucose and lactate. As expected, fractional contribution in upper glycolysis (x-phosphoglycerate and PEP) was almost exclusively from glucose in both iPSCs and MNs compared with lactate, suggesting that gluconeogenesis did not take place in both cell types. Surprisingly, in iPSCs only half of the carbons in glycolysis were labelled after 48 h of ^13^C_6_-glucose incubation, which might indicate a yet unknown carbon source. In both iPSCs and MNs, labelled glucose was metabolised to lactate, as indicated by the presence of glucose-derived ^13^C in lactate, but this tended to be higher in iPSCs (*p* = 0.06). At the same time, MNs consistently showed higher fractional labelling of TCA intermediates from both glucose or lactate (Fig. 2a). These data show that iPSCs have a preference towards producing lactate from pyruvate, whereas MNs have a more oxidative metabolic profile shunting pyruvate into the TCA cycle.

In order to characterise this metabolic switch in more detail, we analysed the fractional abundance of individual isotopologues (Fig. 2b). Glucose can enter the TCA cycle via two main routes, which are via pyruvate dehydrogenase (PDH) or via pyruvate carboxylase (PC). While PDH generates ^13^C m + 2 isotopologues of the TCA cycle intermediates, increased ^13^C m + 3 isotopologues of specific TCA cycle intermediates points towards increased PC activity^31^. Even though PC is traditionally considered insignificant compared with PDH in neurons^32^, PC has been observed in neurons both in vivo and in vitro^33,34^. Our data suggest that MNs have significantly higher PC-dependent activity (based on m + 3 enrichment of TCA cycle intermediates) from both glucose and lactate-derived pyruvate compared with iPSCs (Fig. 2b). In contrast, relative PDH activity (based on m + 2 enrichment of TCA cycle intermediates) from glucose-derived pyruvate remained similar in iPSCs and MNs (Fig. 2b). Remarkably, MNs did show raised PDH activity when carbons were derived from lactate (Fig. 2b). In fact, differences in fractional labelling of TCA intermediates between iPSCs and MNs were much more pronounced for lactate (over twofold increase in fractional label) compared with glucose (30% increase; Fig. 2b).

Taken together, these results show that motor neuron differentiation profoundly rewires metabolic routes by which lactate and glucose are catabolized and this in a substrate specific way.

### Mitochondrial and glycolytic flux in human iPSCs and MNs

**Fig. 3:**
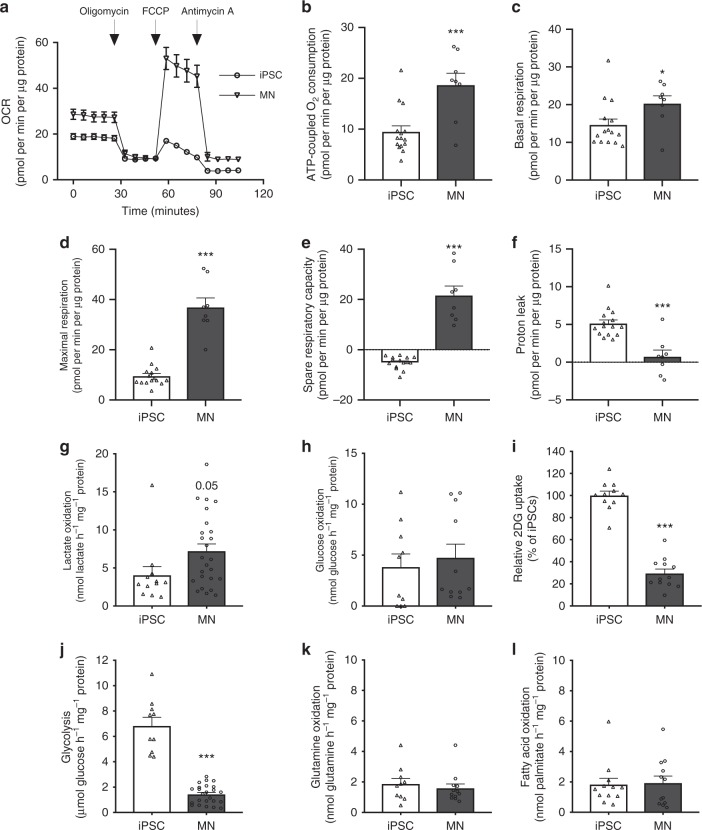
Mitochondrial respiration and metabolic flux in human iPSCs and MNs. **a** Overview of the oxygen consumption rate (OCR) throughout the mitochondrial respiration test in human iPSCs and motor neurons. Arrows indicate the time when mitochondrial inhibitors where added to the media to assess respiratory parameters. **b** ATP-coupled oxygen consumption was determined by inhibiting ATP synthase using oligomycin. **c** Basal respiration in iPSCs and motor neurons. **d** Maximal respiration was assessed following mitochondria uncoupling by FCCP. **e** Spare respiratory capacity was determined by subtracting basal respiration from maximal respiration in iPSCs and motor neurons. **f** Proton leakage in both cell types was determined after inhibiting complex III via antimycin-A. **g** Lactate oxidation in iPSCs and motor neurons. **h** Glucose oxidation in iPSCs and motor neurons. **i** Glucose uptake in iPSCs and motor neurons. **j** Glycolytic flux in iPSCs and motor neurons. **k** Glutamine oxidation in iPSCs and motor neurons. **l** Fatty acid oxidation in iPSCs and motor neurons. **p < 0.01, ***p < 0.0001. Statistical analyses were performed by two-tailed *t* tests to compare iPSCs and motor neurons in panel **b**–**l**. The data represent mean ± s.e.m. from four control cell lines from three independent experiments, with individual data points shown. Source data are provided as a source data file. iPSC induced pluripotent stem cell, MN motor neuron, OCR oxygen consumption rate, FCCP carbonyl cyanide‐4‐(trifluoromethoxy) phenylhydrazone, 2DG 2-deoxy-glucose

As our metabolomics studies indicate altered substrate fate in human iPSCs versus MNs, we investigated whether MNs indeed have a higher oxidative potential by assessing cellular respiration. An overview of the oxygen consumption rate (OCR) throughout the mitochondrial respiration test is provided in Fig. 3a. In agreement with our metabolomics data, ATP-coupled oxygen consumption was on average 96% higher in MNs (Fig. 3b; *p* < 0.01), even though the increase in basal respiration in MNs was limited (Fig. 3c). Moreover, MNs exhibited a marked upregulation of their oxidative potential as indicated by the increase in OCR after mitochondrial uncoupling (Fig. 3d, e; *p* < 0.0001). Additionally, the coupling of ATP synthesis to substrate oxidation was consistently more efficient in MNs compared with iPSCs, as shown by reduced proton leakage in MNs (Fig. 3f; *p* < 0.0001).

To evaluate whether the enhanced oxidative potential of MNs also resulted into increased oxidation of specific energy substrates within the TCA cycle, we performed substrate tracing experiments using radiolabelled glucose, lactate, palmitate and glutamine. In agreement with our ^13^C tracer data, we could detect increased lactate oxidation in MNs (Fig. 3g; *p* = 0.05). The increase in glucose oxidation rate was far less pronounced and failed to reach statistical significance (Fig. 3h). Interestingly, glucose uptake and glycolytic rate were lower in MNs compared with iPSCs (Fig. 3i, j; *p* < 0.0001), which illustrates that MN differentiation is associated with a shift from glycolytic to oxidative metabolism in MNs. As recent in vitro and ex vivo studies have indicated that rat cortical neurons can readily oxidise several other energy substrates^34^, we also assessed flux rate of glutamine and palmitate oxidation in our MNs. We could also detect glutamine and fatty acid oxidation in MNs. However, flux through these pathways was not different between iPSCs and MNs (Fig. 3k, l).

Collectively, our results are the first thorough study on metabolic rewiring upon MN differentiation. We show that iPSCs switch from a glycolytic to an oxidative metabolic state when they differentiate into functional MNs. Our findings add an extra dimension to this traditional concept as we show that MNs mainly use lactate to fuel the increase in oxidative metabolism. Moreover, we show that the routes of metabolic rewirement are energy substrate dependent.

### Mutant FUS does not change MN mitochondrial morphology and respiration

**Fig. 4:**
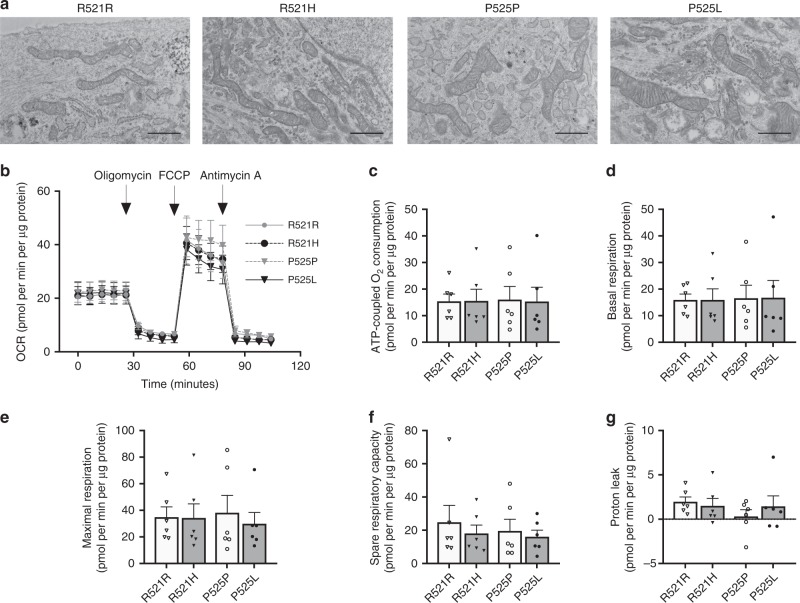
Mitochondrial morphology and respiration in MNs derived from FUS–ALS patients and isogenic controls. **a** Representative transmission electron microscope (TEM) images of the mitochondria from patient (R521H, P525L) and isogenic control (R521R, P525P) motor neurons from three independent experiments. TEM operated at 80 kV at ×10.000 magnification. Scale bar, 1 μm. **b** Overview of the oxygen consumption rates (OCR) throughout the mitochondrial respiration test in patient and isogenic control motor neurons. Arrows indicate the time when mitochondrial inhibitors were added to the media to assess respiratory parameters. **c** ATP-coupled oxygen consumption was determined by inhibiting ATP synthase using oligomycin. **d** Basal respiration in patient and isogenic control motor neurons. **e** Maximal respiration was assessed following mitochondrial uncoupling by FCCP. **f** Spare respiratory capacity was determined by subtracting basal respiration from maximal respiration in patient and isogenic control motor neurons. **g** Proton leakage was determined after inhibiting complex III via antimycin-A. The data represent mean ± s.e.m. from six independent experiments, with individual data points shown. Statistical analyses in panel **c**–**g** were performed by one-way ANOVA with post hoc two-tailed *t* tests between cell lines (*p* > 0.05). Dark grey bars indicate FUS-patient cell lines. Light grey bars indicate isogenic controls from these patients. Source data are provided as a source data file. FCCP carbonyl cyanide‐4‐(trifluoromethoxy) phenylhydrazone

Next, we investigated whether ALS-causing FUS mutations alter energy metabolism in human MNs. While we previously reported that mitochondrial morphology is unaltered in FUS-mutant lines compared to healthy MNs^15^, recent data showed that FUS overexpression impairs mitochondrial structure^21,35^. Moreover, mitochondrial dysfunction is a clinical hallmark of ALS (for a review see:^36^). Therefore, we assessed the direct effect of FUS–ALS causing point mutations on mitochondrial morphology by using transmission electron microscopy in FUS lines and isogenic controls. We did not detect obvious changes in mitochondrial intermembrane space, cristae patterns or overall mitochondrial shape between mutant FUS and corrected MNs (Fig. 4a; Supplementary Fig. 3). In order to determine mitochondrial function, we measured cellular respiration at baseline and after injection of different mitochondrial inhibitors. There were neither significant differences in OCR (Fig. 4b) nor in any individual respiratory parameter (Fig. 4c–g) between FUS-mutant and corrected MNs. Our data show that ALS-mutant FUS did neither affect mitochondrial morphology nor function in iPSC-derived MNs.

### Mutant FUS does not change MN mitochondrial and glycolytic metabolism

**Fig. 5:**
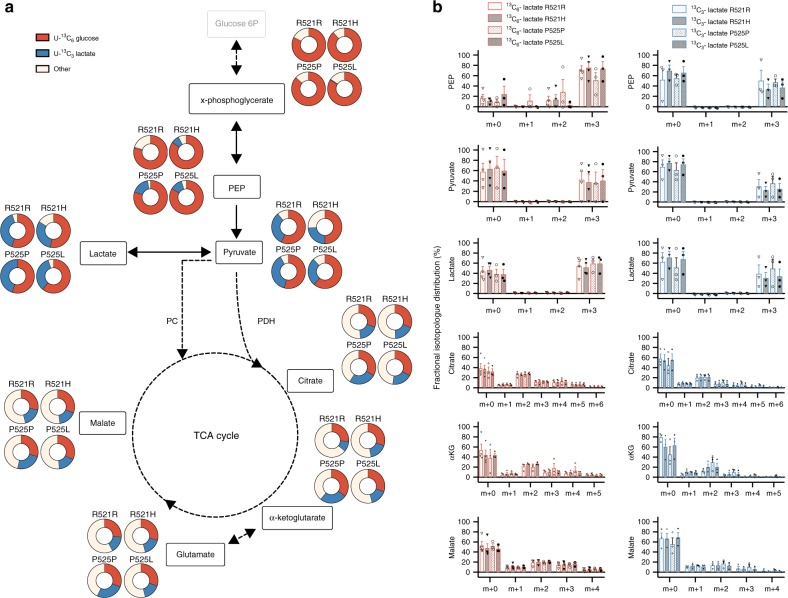
Metabolic fate of glucose and lactate in MNs derived from FUS–ALS patients and isogenic controls. **a** Fractional contribution of uniformly labelled ^13^C glucose (red) and uniformly labelled ^13^C lactate (blue) to metabolites from glycolysis and TCA cycle in patient (R521H or P525L) and isogenic control (R521R or P525P) motor neurons. **b** Relative abundance of each isotopologue for specific metabolites in patient and isogenic control motor neurons. The data represent mean ± s.e.m. from three independent experiments, with individual data points shown. Two-way ANOVA analysis with Tukey post hoc test was used to compare FUS-patients and isogenic controls, but did not show differences (*p* > 0.05). Dark grey bars indicate FUS-patient cell lines. Light grey bars indicate isogenic controls from these patients. Bar fill patterns show the corresponding patient and isogenic control. Thin black arrows represent direct metabolic pathway connections; dashed arrows indicate that additional intermediate reactions are involved in a metabolic pathway. Source data are provided as a source data file. ^13^C_6_-glucose uniformly labelled glucose, ^13^C_3_-lactate uniformly labelled lactate, PEP phosphoenolpyruvate, PC pyruvate carboxylase, PDH pyruvate dehydrogenase, αKG alpha-ketoglutarate, TCA tricarboxylic acid

**Fig. 6:**
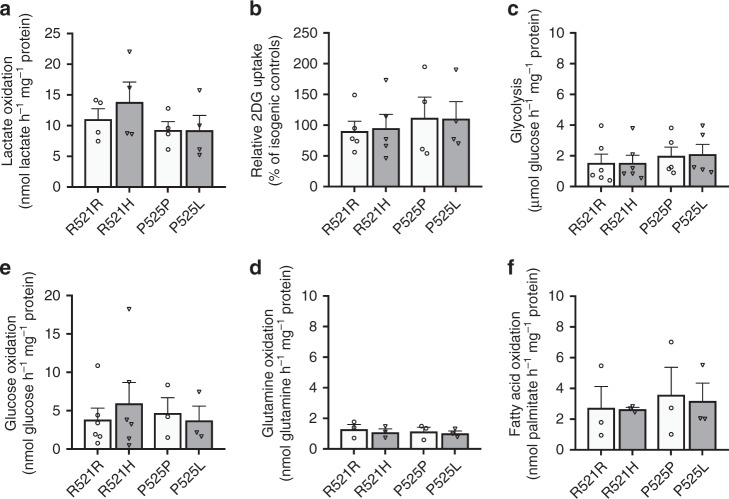
Mitochondrial and glycolytic flux in MNs derived from FUS–ALS patients and isogenic controls. **a** Lactate oxidation in patient (R521H or P525L) and isogenic control (R521R or P525P) motor neurons. **b** Glucose uptake in patient and isogenic control motor neurons. **c** Glycolytic flux in patient and isogenic control motor neurons. **d** Glucose oxidation in patient and isogenic control motor neurons. **e** Glutamine oxidation in patient and isogenic control motor neurons. **f** Fatty acid oxidation in patient and isogenic control motor neurons. The data represent mean ± s.e.m. from at least three independent experiments, with individual data points shown. Statistical analyses were performed by one-way ANOVA with post hoc two-tailed *t* tests between cell lines (*p* > 0.05). Dark grey bars indicate FUS-patient cell lines. Light grey bars indicate isogenic controls from these patients. Source data are provided as a source data file. 2DG 2-deoxy-glucose

Previous reports showed the importance of lactate as a MN energy source^37^ and implicated the downregulation of the lactate transporter MCT1 in ALS pathophysiology^38,39^. Moreover, while the expression of glycolytic enzymes was reduced in ALS patient fibroblasts^40,41^ and motor cortex^42,43^, glycogen storage is increased in the spinal cord from mutant SOD1 mice and autopsied ALS patients^13^. As these results suggest that defects in lactate and glucose catabolism could be relevant to ALS pathophysiology, we performed ^13^C tracer analysis using either ^13^C_3_-lactate or ^13^C_6_-glucose. These experiments revealed no FUS-related changes in the fractional contribution from glucose or lactate to any glycolytic or TCA metabolite (Fig. 5a). When analysing ^13^C isotopologues in detail, no differences were found, indicating that FUS–ALS causing mutations do not rewire lactate and/or glucose metabolism in cultured human MNs (Fig. 5b). In addition to this, we performed radioactive tracer experiments to assess glucose uptake and flux rates through glycolysis and glucose, lactate, palmitate and glutamine oxidation. In agreement with our other findings, FUS mutations did not alter flux rate of any of these critical pathways (Fig. 6a–g). Since FUS mutations lead to a progressive axonal transport defect in MNs^15^, we also evaluated whether metabolic changes present at later stages. To do so, we used motor neurons derived from the most affected ALS patient (P525L), but could not detect any metabolic change between P525L and its isogenic control (Supplementary Fig. 4). Altogether, our data clearly show that ALS-causing point mutations in FUS do not affect the flux though main metabolic pathways in human MNs. As a consequence, it is unlikely that impaired energy metabolism is the main driver of axonal transport defects and hypoexcitability previously observed in these cells^15–18^.

## Discussion

Dysfunctional energy metabolism is linked with ALS pathophysiology^5^. Yet, metabolic properties of human MNs are barely studied, and it is unknown whether ALS MNs differ from healthy MNs. We used stem cell technology and state-of-the-art metabolic tools such as ^13^C tracer analysis and radioactively labelled substrate tracing to investigate this in detail. We discovered that human iPSCs undergo a lactate oxidation fuelled prooxidative metabolic switch when they differentiate into functional MNs. At the same time, they rewire metabolic routes to import pyruvate into the TCA cycle in an energy substrate-specific manner. Combining these tools with patient-derived MNs and their CRISPR/Cas9-corrected counterparts, we show that different ALS causing mutations in FUS did not affect the energy metabolism of cultured human MNs.

Energy metabolism is considered to be an important regulator for the acquisition of stemness^44,45^, as well as during differentiation into specialised cell types^46^. While a shift from a glycolytic to an oxidative metabolic state during the differentiation of primary mouse or iPS cells to cortical neurons was recently shown^47,48^, evidence supporting the presence of a prooxidative shift upon MN differentiation is limited. Moreover, substrate-specific changes in flux or rewiring of metabolic pathways underlying this switch in (motor) neurons are unknown. Therefore, we first characterised the metabolic profile of healthy iPSC-derived MNs. Using multiple iPSC lines, we found that glucose uptake and glycolytic flux severely decreased, while lactate oxidation increased upon differentiation into MNs. In support of this, we also observed increased fractional contribution of both lactate and glucose to TCA intermediates. Furthermore, our MNs showed an increased ATP-coupled oxygen consumption rate. Another study did not detect an increase in ATP-coupled respiration after three weeks of MN differentiation^49^. As the shift towards an oxidative metabotype gets more pronounced throughout differentiation in cortical neurons^48^, this might indicate that our 28 -day long differentiation protocol yields more mature MNs. Indeed, neuronal maturity is also underscored by the presence of elongated, cristae-rich mitochondria in our MN cultures^47,50,51^. Cellular respiration analysis indicated that besides the increase in oxygen consumption, a major factor discriminating iPSCs from MNs was their respiratory reserve. This suggests that iPSCs already use their full oxidative potential at baseline while MNs have the capacity to significantly upregulate their oxidative metabolism. This is in line with their biological function. MNs need to upregulate oxidative metabolism substantially during short periods of time as synaptic transmission largely relies on mitochondrial energy provision^52^. In contrast, proliferation requires continuous mitochondrial respiration to provide sufficient electron acceptors for generating new biomass^53,54^. Whether there is a similar capacity to upregulate glycolysis in MNs, as was recently shown in cortical neurons^55^, or which energy substrate is preferentially oxidised by MNs during increased energy demand^30^, remain open questions. Notably, while differentiation upregulated the influx of both glucose- and lactate-derived carbons into the TCA cycle, differences in lactate were more pronounced compared to differences in glucose. This was supported by a robust increase in lactate oxidation in differentiated MNs, while the increase in glucose oxidation was less pronounced and failed to reach significance. These data indicate that there is a more flexible rewiring of lactate metabolism during MN differentiation compared with glucose metabolism. Next to glycolysis, glucose oxidation and lactate oxidation, we could also consistently detect glutamine and fatty acid oxidation. These findings are in line with recent in vitro studies, showing that rat cortical neurons can oxidise energy substrates besides lactate and glucose^34^. In addition to in vivo confirmation, future studies should be performed to investigate the functional relevance of fatty acid and glutamine oxidation in MNs in health as well as in disease.

Besides a global shift in metabolic state, we show that iPSCs rewire key metabolic routes upon differentiation into MNs. We found that MNs upregulate pyruvate entry into the TCA cycle via PC. This was true for both glucose and lactate-derived pyruvate. In contrast, MNs only upregulated the utilisation of lactate-derived pyruvate for PDH. While the functional relevance of PC in neurons is debated^56^, our data support an active role for PC in controlling pyruvate entry into the TCA cycle in MNs. This is likely for different reasons. First, the importance of PC could result from increased lactate dehydrogenase activity which is required to convert lactate to pyruvate and therefore to sustain increased lactate oxidation. As lactate dehydrogenase generates NADH, a powerful allosteric PDH inhibitor^56^, PC would be essential to sustain active TCA cycling under conditions where lactate oxidation, and thereby NADH generation, is increased, such as in MNs. Second, PC could be particularly vital for MNs to compensate for losses of α-ketoglutarate as it is constantly drained from the TCA cycle for the generation of the α-ketoglutarate-derived neurotransmitters glutamate and GABA^57^. It remains unknown why only lactate—and not glucose-dependent PDH flux—was higher in MNs compared with iPSCs. However, it is tempting to speculate that the clear distinction between glucose and lactate metabolic rewiring is caused by the high degree of metabolic compartmentalisation in MNs^58,59^. Indeed, it becomes increasingly clear that different neuronal compartments have distinctive energy requirements^58^. Moreover, different neuronal processes depend on specific metabolic processes^60^. Hence, metabolic compartmentalisation in MNs could also be linked to the metabolism of specific energy substrates.

Given the amount of circumstantial evidence linking energy metabolism to ALS, the absence of metabolic alterations in FUS–ALS MNs was unexpected. Most studies which reported metabolic dysfunction in FUS–ALS have used models that overexpress FUS. Mitochondrial vacuolisation was observed in MNs from mice^10^, flies and HEK cells^21^ overexpressing mutant or wild-type human FUS. Nevertheless, inclusions containing disorganised mitochondria were also observed in the *postmortem* spinal cord of juvenile ALS patients carrying the P525L mutation^61^. In addition, we and others previously showed that mutant FUS directly causes defects in axonal transport^15,16^ and synaptic activity^15–18^ in cultured human MNs. The high dependence of both processes on continuous energy provision suggests that dysfunctional metabolism could cause these ALS-related phenotypes. However, we did neither observe abnormalities in the morphology of mitochondria in our P525L-mutant MNs nor in another ALS patient line carrying the R521H mutation. Mitochondria in FUS–ALS patient-derived MNs exhibited an elongated morphology and clear cristae structure, indicative of proper function^62^. Although we cannot exclude subtle morphological changes, FUS mutations also did not affect ATP-coupled oxygen consumption, maximal or reserve respiratory capacity nor the oxidation of lactate, glucose, glutamine or fatty acids. This is in apparent contrast with recent work which showed impaired mitochondrial ATP generation upon inducible overexpression of FUS^21^. While overexpression of P525L-mutant FUS indeed affected the activity of dimerised ATP synthase complexes more than overexpression of WT FUS, the reduction of mitochondrial ATP synthesis was similar upon overexpression of WT or P525L-mutant FUS. This suggests that overexpression of FUS per se, rather that the pathogenic effect of the mutation, could affect ATP synthesis^20,21^.

Our data do not imply that energy metabolism does not play a role in ALS nor that therapies targeting energy metabolism could not be beneficial to ALS patients. Indeed, ALS is a complex disease in which various cell types interact to induce MN degeneration^63^. For example, oligodendrocytes, which are vital to metabolically support MNs, are implicated in ALS pathogenesis^38^. Moreover, reprogramming of somatic cells to iPSCs erases the epigenetic signature which is increasingly recognised as a vital component in ALS aetiology^64,65^. The results from patient-derived MNs should therefore be interpreted with the limitations of the current model in mind. Future studies using more complex patient-derived culture systems^66^, which better mimic the MN microenvironment by for example including glial cells, could be required to address these outstanding questions. Despite these limitations, iPSC technology offers the intriguing opportunity to study ALS using patient-derived MNs without the need to overexpress the transgene. In addition, we and others showed that several aspects of FUS–ALS are recapitulated in iPSC-derived MNs further underscoring the value of this model^23^.

In conclusion, our study represents the first thorough characterisation of the energy metabolism of human iPSC-derived MNs. In addition, we show that different FUS point mutations do not affect energy metabolism in MNs derived from FUS–ALS patients. Since we have previously shown multiple ALS-related phenotypes in this model, our current results imply that metabolic dysfunction is not the underlying cause of these phenotypes.

## Methods

### iPSC culture

Human iPSCs were maintained on GeltrexR (A1413302, Gibco) in Essential 8 medium (A1517001, Gibco) supplemented with penicillin–streptomycin. Colonies were routinely passaged with 0.5 mM EDTA (15575–020, Invitrogen) in Dulbecco’s phosphate-buffered saline (DPBS). Cultures were routinely analysed for mycoplasma contamination by PCR. Seven days before the experimental day, iPSCs were passaged to reach 70–80% confluence during the experiment. To assess lactate catabolism, Essential 8 medium was supplemented with 2 mM lactate (71718, Sigma) 72 h prior to the experiments. For reasons of consistency, 2 mM lactate was maintained in all metabolic experiments.

### Differentiation of iPSCs to MNs

MNs were differentiated from iPSCs as described before^15,27^. Briefly, iPSC clones were suspended and transferred from a six-well plate into a T-25 flask with neuronal basic medium (a 1:1 mixture of Neurobasal medium and DMEM/F12 medium, with N2 and B27 without vitamin A), using collagenase type IV digestion to form embryoid bodies. After 2 days incubation with 5 μM ROCK Inhibitor (Y-27632, Merck Millipore), 40 μM TGF-β inhibitor (SB 431524, SB, Tocris Bioscience), 0.2 μM bone morphogenetic protein inhibitor (LDN-193189, LDN, Stemgent) and 3 μM GSK-3 inhibitor (CHIR99021, CHIR, Tocris Bioscience), suspended embryoid bodies were incubated with neuronal basic medium containing 0.1 μM retinoic acid (RA, Sigma) and 500 nM Smoothened Agonist (SAG, Merck Millipore) for 4 days. Cells were subsequently incubated for 2 days in a neuronal basic medium containing RA, SAG, 10 ng/ml brain-derived neurotrophic factor (BDNF, Peprotech), and 10 ng/ml glial cell-derived neurotrophic factor (GDNF, Peprotech). At day 9 of differentiation, cell spheres were dissociated into single cells using 0.05% trypsin (Gibco) for 20 min at 37 °C. After cell counting, a defined number of cells were seeded in poly-L-ornithine (100 µg/ml) and laminin (20 μg/ml)-coated plates and incubated for 5 days in a neuronal basic medium containing RA, SAG, BDNF, GDNF and 10 μM inhibitor of γ-secretase (DAPT, from Tocris Bioscience), and then incubated for 2 days in a neuronal basic medium containing BDNF, GDNF and 20 μM DAPT. For MN maturation, cells were kept for 12 days in neuronal medium supplemented with BDNF, GDNF and ciliary neurotrophic factor (CNTF; 10 ng/ml each, Peprotech). Media were changed every other day by replacing half of the medium. All experiments were performed during the fourth week of differentiation. Seventy-two hours before metabolic experiments, MNs were cultured in mature MN media containing 5 mM glucose and 2 mM lactate, concentrations resembling the in vivo situation^67^.

### RNA sequencing

RNA sequencing was performed by the Nucleomics Core Facility (VIB, Leuven, Belgium). RNA was isolated using an RNeasy kit (Qiagen). From extracted RNA, libraries were made using the Illumina TruSeq Stranded mRNA Library protocol. These libraries were sequenced on an Illumina NextSeq 500 paired-end 75 bp and yield an average of 90.2 million reads per sample (range 75.6–106.9). To estimate the expression of the transcript of every sample, reads were counted using Salmon (v0.8.1)^68^ against the ensembl transcript for the human reference genome hg38. Gene expression from the protein coding transcripts was then estimated using the tximport function the R-package tximport (v1.6.0)^69^.

### Immunocytochemistry

Cells plated on coverslips were fixed in 2% paraformaldehyde (PFA) for 20 min at room temperature and were washed with PBS. Permeabilization and blocking was done for 30 min using PBS containing 0.1% Triton X-100 (Acros Organics) and 5% normal donkey serum (Sigma) for 1 h. Cells were incubated overnight at 4 °C in blocking buffer (2% donkey serum) containing the different primary antibodies (Supplementary Table 2). After washing with PBS containing 0.1% Triton X-100, cells were incubated with secondary antibodies (Invitrogen) for 1 h at room temperature. Fluorescent micrographs were captured using a Zeiss Axio Imager M1 microscope equipped with a monochrome AxioCam Mrm camera (fluorescence, Carl Zeiss). Images were analysed using ImageJ.

### Electrophysiological recordings

Whole-cell patch-clamp recordings were used to determine the functionality of iPSC-derived MNs. Experiments were performed in a recording chamber which was continuously perfused with artificial cerebrospinal fluid (aCSF) at room temperature. ACSF contained (in mM) 140 NaCl, 5 KCl, 2 CaCl_2_, 2 MgCl_2_, 10 HEPES and 12 glucose (pH adjusted to 7.4 with NaOH; ~300 mOsm). Cells were visualised by an inverted Olympus IX73 microscope equipped with a ×40 objective. Recording patch pipettes were pulled from borosilicate glass using a vertical PIP6 micropipette puller (HEKA Elektronik, Lambrecht/Pfalz, Germany) and filled with an internal solution containing (in mM): 120 K-gluconate, 20 KCl, 1 MgCl2, 10 HEPES, 0.2 EGTA, 0.3 Na-GTP, 5 NaCl, 4 Mg-ATP (pH adjusted to 7.3 with KOH; ~290 mOsm). Firing properties of MNs were assessed in current clamp mode. Action potentials were evoked by applying 1-s current pulses ranging from −50 pA to + 60 pA in 10-pA steps. The membrane potential was clamped at −65 mV between current pulses. Spontaneous action potentials were recorded without current injection. Voltage-gated Na^+^ and K^+^ channels were recorded in voltage–clamp mode by applying 200-ms voltage pulses from −80 mV to + 50 mV in 10-mV steps. An online P4 leak subtraction protocol was applied during the acquisition of voltage-gated currents. Signals were acquired, filtered (at 2.8 kHz) and digitised (at 20 kHz) using an EPC10 USB amplifier and PatchMaster software (HEKA Elektronik). The liquid junction potential of 13.8 mV was corrected off-line. Action potentials were detected using Stimfit software; current amplitudes were quantified by PatchMaster software.

### Liquid chromatography mass spectrometry

iPSCs and MNs were cultured in mature MN media containing either 5 mM uniformly labelled ^13^C-glucose (Cambridge Isotope Laboratories) with 2 mM unlabelled lactate or 2 mM uniformly labelled ^13^C-lactate (Cambridge Isotope Laboratories) with 5 mM unlabelled glucose. Uniform labelling of tracers was assured by isotopologue analysis (Supplementary Fig. 5). Cells were incubated with ^13^C-labelled media for 48 h before metabolite extraction to allow isotopic steady state to occur in both glycolytic and TCA intermediates^31^. Polar metabolites were extracted using 150 μL of a 80% methanol (in water) extraction buffer containing 2 μM of deuterated (d27) myristic acid as internal standard. Following extraction, precipitated proteins and insolubilities were removed by centrifugation at 20.000×*g* for 20 min at 4 °C. The supernatant was transferred to the appropriate mass spectrometer vials. Measurements were performed using a Dionex UltiMate 3000 LC System (Thermo Scientific) in-line connected to a Q-Exactive Orbitrap mass spectrometer (Thermo Scientific). In all, 15 μl of sample was injected and loaded onto a Hilicon iHILIC-Fusion(P) column (Achrom). A linear gradient was carried out starting with 90% solvent A (LC-MS grade acetonitrile) and 10% solvent B (10 mM ammoniumacetate pH 9.3). From 2 to 20 min, the gradient changed to 80% B and was kept at 80% until 23 min. Next, a decrease to 40% B was carried out to 25 min, further decreasing to 10% B at 27 min. Finally, 10% B was maintained until 35 min. The solvent was used at a flow rate of 200 μl/min, the columns temperature was kept constant at 25 °C. The mass spectrometer operated in negative ion mode, settings of the HESI probe were as follows: sheath gas flow rate at 35, auxiliary gas flow rate at 10 (at a temperature of 260 °C). Spray voltage was set at 4.8 kV, temperature of the capillary at 300 °C and S-lens RF level at 50. A full scan (resolution of 140.000 and scan range of m/z 70–1050) was applied. For the data analysis, we used an in-house library, and metabolites of interest were quantified (area under the curve) using the XCalibur 4.0 (Thermo Scientific) software platform. Correction of natural abundance and calculations of fractional contribution was carried out using an in-house software platform.

### Metabolic flux experiments

Metabolic fluxes were measured using radiolabelled glucose, lactate, palmitate and glutamine based on previously established methods^70^. Specific activity was kept identical between E8 versus mature MN media for all experiments.

Glycolysis: Cells were incubated for 6 h in medium containing 0.89 µCi D-[5-^3^H]-glucose (15.2 Ci/mmol; Perkin Elmer) per mmol cold glucose. Glycolytic flux was measured by the rate of ^3^H_2_O production from D-[5-^3^H]-glucose^70^. Briefly, after 6 h incubation, the culture medium was transferred into glass vials sealed with rubber stoppers. ^3^H_2_O was captured in hanging wells containing a H_2_O-soaked Whatman paper over a period of 48 h at 37 °C. Radioactivity was determined by liquid scintillation counting.

Fatty acid oxidation: Fatty acid oxidation was measured by the rate of ^3^H_2_O production in the supernatant, during 6 h incubation of cells in medium, supplemented with 100 µM unlabelled palmitate and 50 µM carnitine (Sigma-Aldrich) and containing 77.99 µCi [9,10-^3^H(N)]-palmitate (53.7 Ci/mmol; Perkin Elmer) per mmol cold palmitate. ^3^H_2_O recovery was performed as described for glycolysis.

Glucose oxidation: Cells were incubated for 6 h in medium containing 0.61 µCi D-[6-^14^C]-glucose (55 mCi/mmol; American Radiolabelled Chemicals) per mmol cold glucose, after which cellular metabolic reactions were stopped by the addition of perchloric acid (PCA). Glucose oxidation was measured by the rate of ^14^CO_2_ production^70^. Briefly, immediately after adding PCA to the medium, wells were covered with 1× hyamine hydroxide-saturated Whatman papers. The released ^14^CO_2_ was captured into the papers overnight at room temperature, and radioactivity was determined by liquid scintillation counting.

Glutamine oxidation: Glutamine oxidation was measured by the rate of ^14^CO_2_ production during 6 h incubation of cells in their respective medium containing 0.94 µCi L-[^14^C(U)]-glutamine (267 mCi/mmol; Perkin Elmer) per mmol cold glutamine. ^14^CO_2_ recovery was performed as described for glucose oxidation.

Lactate oxidation: Lactate oxidation was measured by the rate of ^14^CO_2_ production during 6 h incubation of cells in their respective medium containing 2.45 µCi L-[^14^C(U)]-lactate (154.8 mCi/mmol; Perkin Elmer) per mmol cold lactate. ^14^CO_2_ recovery was performed as described for glucose oxidation.

^14^C-2DG uptake. Uptake was measured by incubating the cells with 500 µl of medium containing 0.56 µCi 2-[^14^C(U)]-deoxy-D-glucose (264 mCi/mmol; Perkin Elmer) per mmol cold glucose for 2 h. Uptake was stopped by adding 1 ml of stop solution (50 mM glucose in icecold PBS). After two subsequent washes with 1 ml of stop solution, cells were lysed in 0.5 ml of 0.1 N NaOH and samples were counted by liquid scintillation counting.

### Oxygen consumption

At day 10 of differentiation, MN progenitors were seeded on Seahorse XF24 tissue culture plates (Seahorse Bioscience Inc.). At day 30 of differentiation or 1 week after passaging for MNs or iPSCs, respectively, oxygen consumption rate (OCR) was measured over intervals of 2 min. In baseline conditions, five consecutive measurements of OCR were done. Thereafter, ATP synthase inhibition was achieved by the injection of oligomycin (12.4 μM, concentration in well 1.2 μM). The drop in OCR reflects the oxygen consumption serving ATP production. Next, the mitochondrial uncoupler carbonilcyanide p-triflouromethoxyphenylhydrazone (FCCP) was injected at 100 μM (concentration in well 10 μM). This forces cells to use their maximal mitochondrial capacity in order to preserve the mitochondrial membrane potential. The increase in OCR thus reflects the reserve electron transporting capacity of mitochondria. As a third injection, the complex III inhibitor antimycin-A was added at 10 μM (concentration in well 1 μM). This shuts down electron transport chain activity and abolishes mitochondrial oxygen consumption, allowing the calculation of proton leakage as the difference in OCR after oligomycin and antimycin-A injection. All mitochondrial inhibitors were obtained from Sigma.

### Transmission electron microscopy

Cells were fixed by adding 1 ml of pre-warmed double strength fixative (8% PFA (Electron Microscopy Services #15714) and 5% glutaraldehyde (Electron Microscopy Services #16220) in 0.1 M sodium cacodylate buffer, pH 7.4 (Electron Microscopy Services #12300)) to 1 ml medium while rotating at room temperature. After 10 min, the solution was replaced by single strength fixative (4% PFA with 2.5% glutaraldehyde in 0.1 M sodium cacodylate buffer). Cells were kept in this fixative until further use at 4 °C.

Next, cells were stained with 1% osmium tetroxide (Electron Microscopy Services #19152) with 1.5% potassium ferrocyanide (Sigma #455989) in ddH_2_O for 60 min, subsequently with 0.2% tannic acid (Electron Microscopy Services #21700)) in ddH_2_O for 30 min and with 1% osmium tetroxide in ddH_2_O for 30 min, followed by overnight incubation in 0.5% uranyl acetate in 25% methanol at 4 °C. The next day, cells were incubated en bloc with Walton’s lead aspartate (0.02 M lead nitrate (Electron Microscopy Services #17900) in 0.03 M sodium aspartate (Sigma #11189), pH 5.5)) for 30 min at 60 °C in dark. After dehydration with increasing concentrations of ethanol, cells were embedded in Agar 100 (Laborimpex) with inverted BEEM-capsules (Laborimpex) and polymerised for 48 h at 60 °C. The glass coverslip was removed by putting the dish for 20 min in DOW CORNING^®^ OS-30 (1020030_FL500G, Mavom). Once the BEEM-capsules were removed, blocks were trimmed and 70 nm sections cutted using a Leica Ultracut S. Sections were collected on slot grids and imaged with a JEOL JEM-1400 Transmission Electron Microscope operated at 80 kV at ×10.000 magnification.

### Statistics

For each experiment, a minimum of three different experiments based on three independent differentiation batches were performed. ‘*n*’ indicates the number of experiments performed. Statistical analysis was done using Graphpad Prism (version v8.0). Two-tailed *t* tests were used to compare iPSCs to MNs. One-way ANOVA with Tukey post hoc test was used to compare: MN marker expression, respiration parameters between FUS-patients and isogenic controls, metabolic fluxes between FUS-patients and isogenic controls. Two-way ANOVA analysis with Tukey post hoc test was used to compare fractional isotopologue distribution patterns between FUS-patients and isogenic controls. *p*-values < 0.05 were considered significant (^$^*p* < 0.10, *p < 0.05, **p < 0.01, ***p < 0.001). The data represent mean ± s.e.m.

### Reporting summary

Further information on research design is available in the Nature Research Reporting Summary linked to this article.

## Supplementary information

Supplementary Information

Peer Review

Reporting Summary

Source Data File

## Data Availability

Transcriptomic data that support the findings of this study have been deposited in European Genome-phenome Archive with the accession codes “EGAD00001005216” or “EGAS00001003785”. The authors declare that the other data supporting the findings of this study are available within the source data file.
