# Supplementary material for: Differentiation but not ALS mutations in FUS rewires motor neuron metabolism
Source: Nat Commun. 2019 Sep 12;10:4147. doi: 10.1038/s41467-019-12099-4 (PMC6742665; doi:10.1038/s41467-019-12099-4)
Supplement: Supplementary file 1 — Supplementary Information [file 41467_2019_12099_MOESM1_ESM.pdf]

Supplementary Information

Differentiation but not ALS mutations in FUS rewires motor neuron metabolism

Vandoorne et al.

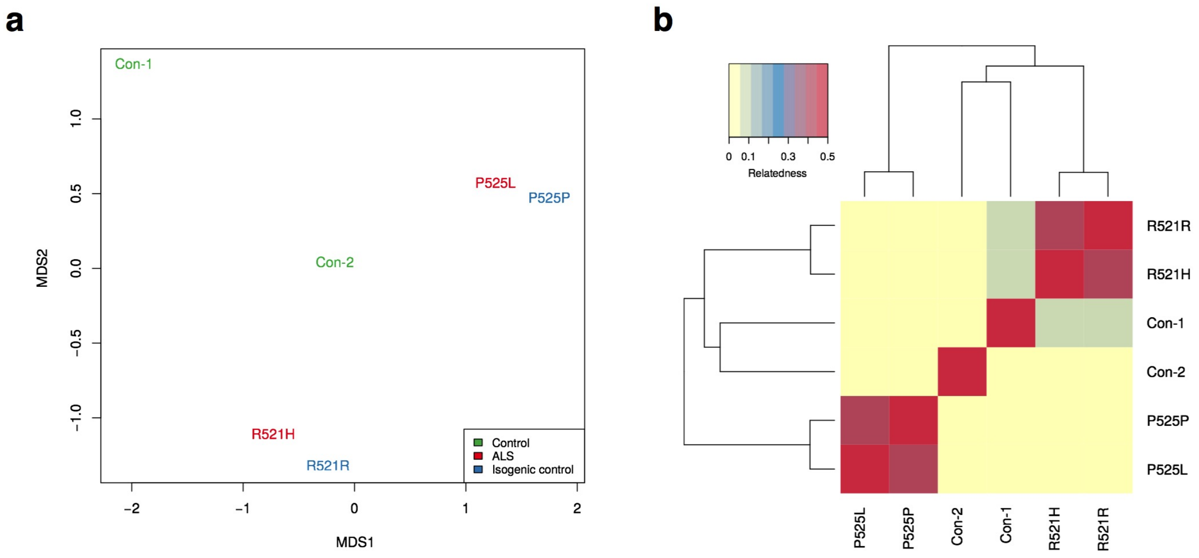

Supplementary Fig. 1 RNA-sequencing analysis of relatedness between different MN lines. **a,** MDS plot Multidimensional scaling plot made by the R-package limma (v3.34.9) using the top 500 autosomal expressed genes. **b,** Relatedness plot. The genetic relatedness between the different samples was calculated using the relatedness2 function of VCFtools (v0.1.15) using variants called by gatk-haplotype of the GATK software suite (v4-4.0b6-0), after mapping raw read on hg38 with decoys using STAR (v2.5.3a). A relatedness score of 0.5 indicate full related samples, while a relatedness score of 0 means that the sample are not related. Raw data are deposited publicly, EGA (EGAS00001003785).

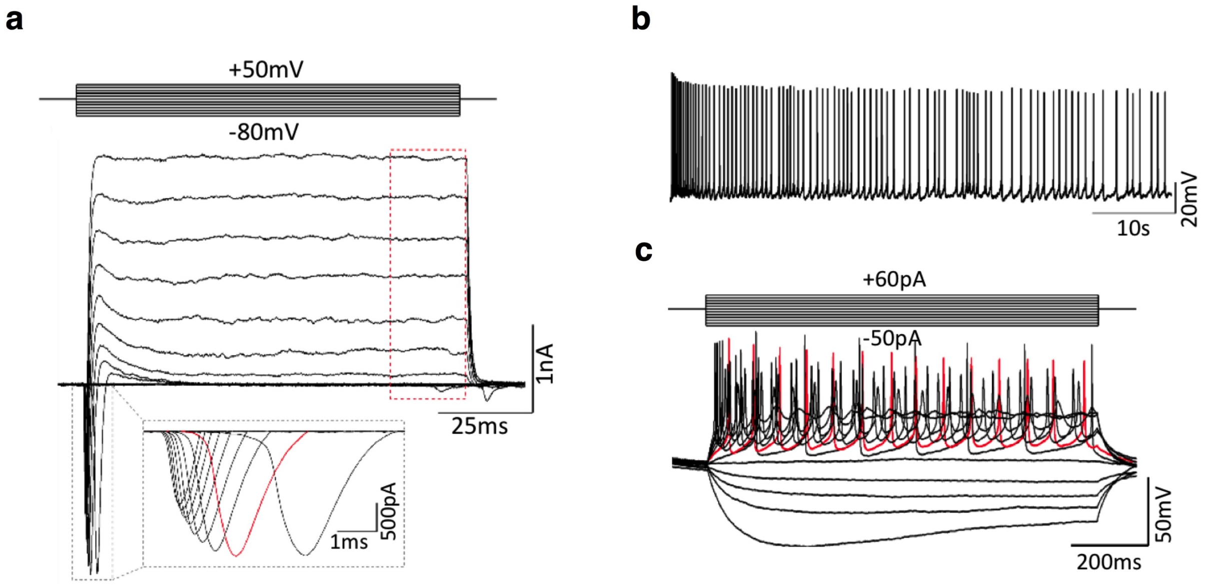

Supplementary Fig. 2 Electrophysiological recordings in Con-1 MNs at the fourth week of differentiation. **a,** Experimental voltage pulse step protocol (top panel) and representative traces of current responses showing voltage-activated in- and outward currents. Outward peak currents were quantified at +20mV during the time course indicated by the red dashed box. The amplitude of the peak inward current (red trace in grey dashed box) was measured for every cell. Inward current density -104±9pA/pF (n=15), outward current density +20mV: 47±6pA/pF (n=13). **b,** Representative recording of spontaneous action potentials, 18/19 measured cells fired spontaneous action potentials (firing frequency: 1.0±0.2Hz). **c,** Experimental current pulse step protocol (top panel) and representative traces of membrane potential responses (lower panel). The maximal amount of action potentials (red trace) in response to a depolarizing current pulse was determined for every cell. In all motor neurons, action potentials could be elicited (maximal firing frequency: 6.7±1.1Hz; n=19). Motor neurons demonstrated an average resting membrane potential of -57±2mV and an average cell capacitance of 26±3pF (n=19). These results are similar to our previous reported findings (Guo et al., Nature communications, 2017). 'n' indicates the number of patched cells. Source data are provided as a source data file.

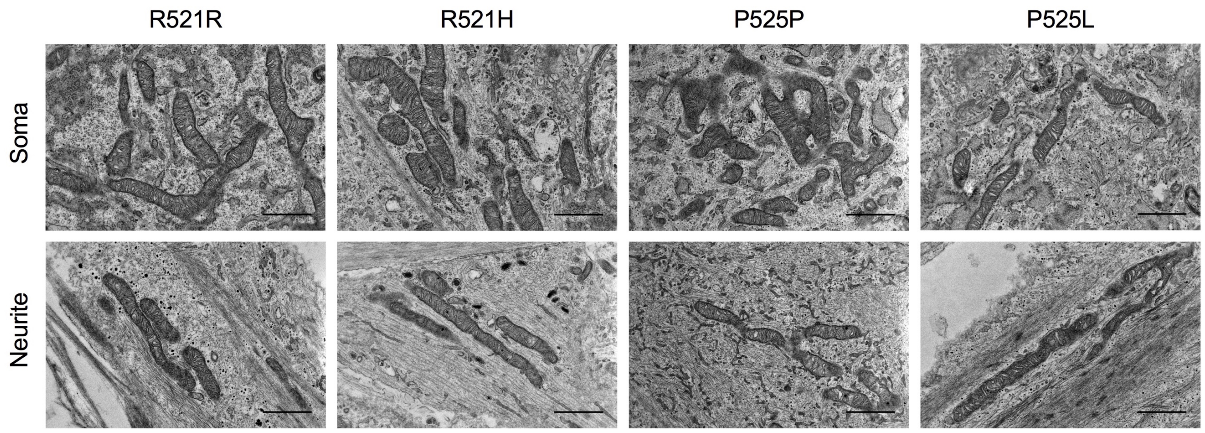

Supplementary Fig. 3. Mitochondrial morphology in MNs from FUS-ALS patients and isogenic controls. Representative transmission electron microscope (TEM) images of mitochondria from patient (R521H, P525L) and isogenic control (R521R, P525P) in the soma or neurites of motor neurons from three independent experiments. TEM operated at 80kV at 10.000x magnification. Scale bar, 1 µm.

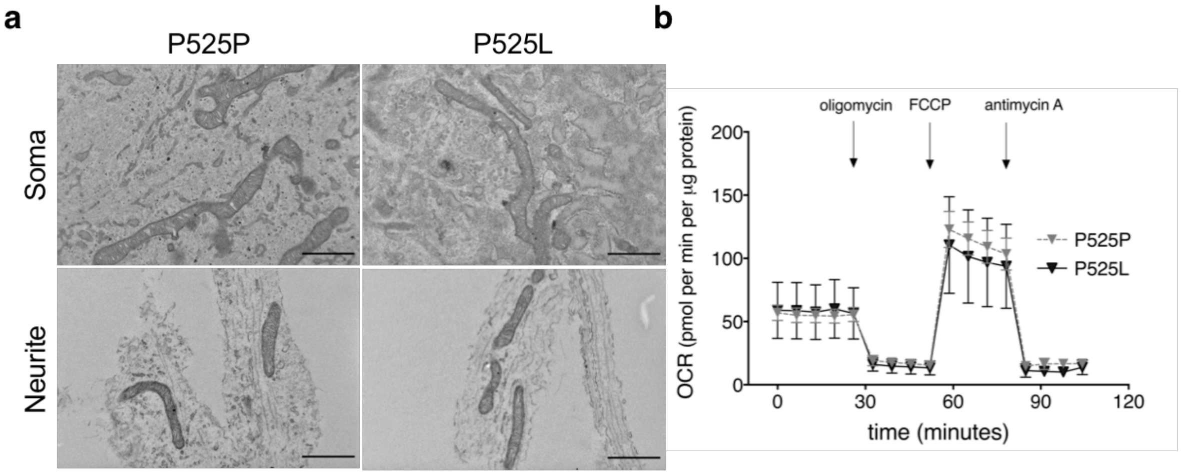

c

		xPG	PEP	pyruvate	lactate	Ala	citrate	αkg	malate	Glu	Asp
P525P $^{13}C_6$ glucose	differentiation 1	92,2%	95,4%	23,8%	23,5%	13,8%	20,2%	13,2%	19,8%	23,8%	19,9%
	differentiation 2	94,5%	97,5%	36,1%	33,5%	25,0%	15,1%	17,0%	25,5%	31,2%	24,1%
	differentiation 3	91,1%	94,7%	44,8%	50,5%	33,4%	34,1%	29,4%	31,2%	37,6%	32,5%
P525L $^{13}C_6$ glucose	differentiation 1	90,2%	95,1%	26,0%	20,8%	16,9%	16,7%	11,9%	18,4%	21,4%	16,9%
	differentiation 2	94,8%	96,4%	37,3%	38,7%	27,3%	27,9%	20,6%	28,0%	33,6%	28,0%
	differentiation 3	91,0%	92,3%	42,7%	46,5%	30,3%	32,8%	28,1%	29,9%	36,4%	30,8%
P525P $^{13}C_3$ lactate	differentiation 1	0,4%	0,1%	37,3%	55,8%	32,1%	31,4%	24,5%	31,8%	39,2%	32,8%
	differentiation 2	0,6%	0,3%	27,1%	42,1%	27,5%	24,6%	20,8%	24,8%	30,3%	25,3%
	differentiation 3	0,4%	0,3%	36,3%	52,9%	36,7%	31,8%	29,5%	29,0%	36,7%	30,9%
P525L $^{13}C_3$ lactate	differentiation 1	0,5%	0,2%	38,6%	54,1%	29,0%	26,0%	19,5%	24,4%	30,0%	24,5%
	differentiation 2	0,6%	0,3%	25,9%	38,7%	28,5%	22,6%	19,3%	21,9%	25,0%	22,5%
	differentiation 3	0,3%	0,7%	47,4%	71,1%	32,9%	37,0%	31,9%	35,1%	45,2%	34,6%

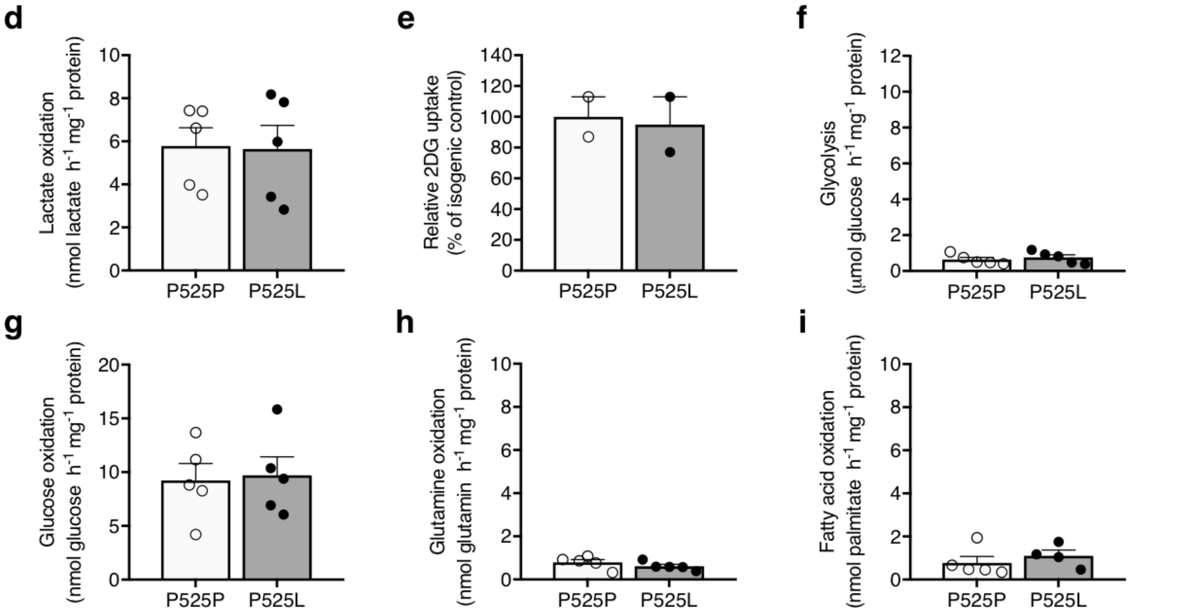

Supplementary Fig. 4. P525L mutant FUS do not affect energy metabolism in patient derived MNs during the seventh week of differentiation. **a,** Transmission electron microscope (TEM) images of mitochondria from patient (P525L) and isogenic control (P525P) motor neurons during the seventh week of differentiation did not reveal gross morphological alterations (n=3). TEM operated at 80kV at 10,000x magnification. Scale bar, 1 µm. **b,** Overview of oxygen consumption rates (OCR) throughout the mitochondrial respiration test in patient (P525L) and

isogenic control (P525P) motor neurons during the seventh week of differentiation. Arrows indicate the time when mitochondrial inhibitors were added to assess respiratory parameters. Statistical analyses were performed by Two-way ANOVA ($p>0.05$; n=3). **c,** Fractional contribution of uniformly labelled ^{13}C glucose and uniformly labelled ^{13}C lactate to metabolites from glycolysis and TCA cycle did not show any differences between patient (P525L) and isogenic control (P525P) motor neurons during the seventh week of differentiation (n=3). **d,** Lactate oxidation (n=5) **e,** glucose uptake (n=2) **f,** glycolysis (n=5) **g,** glucose oxidation (n=5) **h,** glutamine oxidation (n=5) and **i,** fatty acid oxidation (n=5) was indifferent in patient (P525L) and isogenic control (P525P) motor neurons during the seventh week of differentiation. Statistical analyses in panel **c-i** were performed by standard t-tests to compare iPSCs and motor neurons ($p>0.05$). Data represents mean ± s.e.m. for at least three independent experiments with individual data points shown. Source data are provided as a source data file. FCCP, carbonyl cyanide-4-(trifluoromethoxy) phenylhydrazone; $^{13}C_6$-glucose, uniformly labelled glucose; $^{13}C_3$-lactate, uniformly labelled lactate; xPG, x-phosphoglycerate; PEP, phosphoenolpyruvate; Ala, alanine; αKG, alpha-ketoglutarate; Glu, glutamate; Asp, aspartate; 2DG, 2-deoxy-glucose.

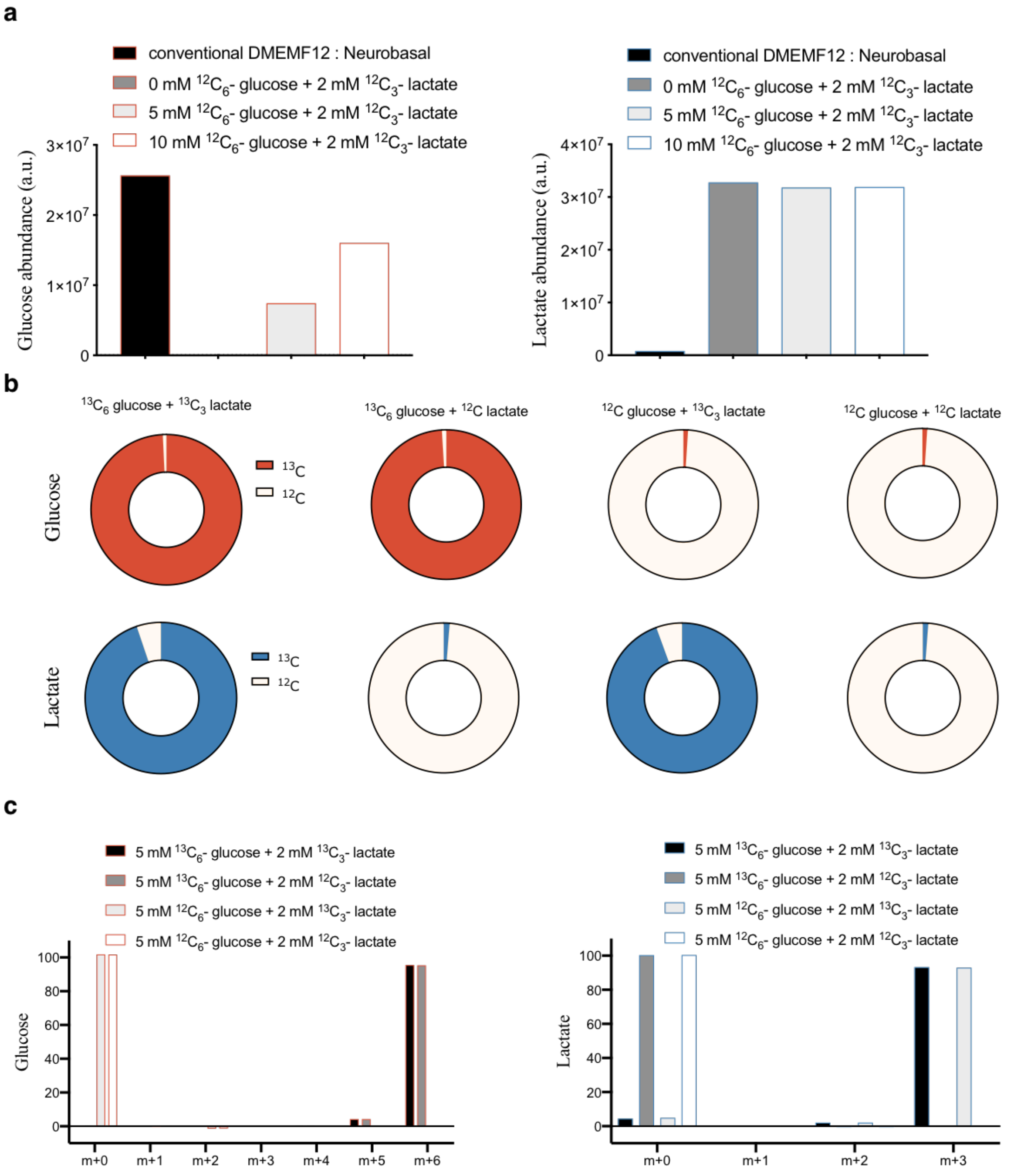

Supplementary Fig. 5. Quality control of glucose free media and ^{13}C isotope tracers. **a,** Abundance of glucose and lactate in culture media to which different concentrations of glucose and lactate were added. Confirming the absence of glucose and lactate from our glucose free media. **b,** Fractional contribution of $^{13}C_6$ glucose (red) and $^{13}C_3$ lactate (blue) to glucose and lactate in culture media containing either 5 mM $^{13}C_6$ or 5 mM ^{12}C glucose with 2 mM $^{13}C_3$ or 2 mM ^{12}C lactate. **c,** Relative abundance of each isotopologue for glucose and lactate in culture media containing 5mM $^{13}C_6$ or ^{12}C glucose with 2mM $^{13}C_3$ or ^{12}C lactate. Results in panel **b,c** confirm uniform labelling of ^{13}C tracers. Source data are provided as a source data file.

Code	ALS mutation	Diagnosis	Gender	Age at biopsy
Con-1	None	/	F	39
Con-2	None	/	M	52
R521H	FUS R521H	FUS-ALS	F	71
R521R	None		F	71
P525L	FUS P525L	FUS-ALS	M	17
P525P	None		M	17

Supplementary Table 1 Overview of human iPSC lines used in this study.

Antibody	Isotype	Dilution	Source
Smi32	Rabit IgG	1/1000	Abcam
Isl1	Rabbit IgG	1/200	Merck Millipore
Tuj1	Mouse IgG	1/500	Abcam
ChAT	Rabbit IgG	1/500	Merck Millipore
Synapsin 1	Rabbit IgG	1/2000	Merck Millipore

Supplementary Table 2 List of antibodies used for immunocytochemistry.
