# Supplementary material for: Differentiation but not ALS mutations in FUS rewires motor neuron metabolism
Source: Nat Commun. 2019 Sep 12;10:4147. doi: 10.1038/s41467-019-12099-4 (PMC6742665; doi:10.1038/s41467-019-12099-4)
Supplement: Supplementary file 3 — Reporting Summary [file 41467_2019_12099_MOESM3_ESM.pdf]

nature research

Corresponding author(s): Prof. dr. Ludo Van den Bosch

Last updated by author(s): Aug 1, 2019

Reporting Summary

Nature Research wishes to improve the reproducibility of the work that we publish. This form provides structure for consistency and transparency in reporting. For further information on Nature Research policies, see Authors & Referees and the Editorial Policy Checklist .

Statistics

For all statistical analyses, confirm that the following items are present in the figure legend, table legend, main text, or Methods section.

n/a	Confirmed	
☐	☒	The exact sample size (*n*) for each experimental group/condition, given as a discrete number and unit of measurement
☒	☐	A statement on whether measurements were taken from distinct samples or whether the same sample was measured repeatedly
☐	☒	The statistical test(s) used AND whether they are one- or two-sided<br>*Only common tests should be described solely by name; describe more complex techniques in the Methods section.*
☒	☐	A description of all covariates tested
☐	☒	A description of any assumptions or corrections, such as tests of normality and adjustment for multiple comparisons
☐	☒	A full description of the statistical parameters including central tendency (e.g. means) or other basic estimates (e.g. regression coefficient) AND variation (e.g. standard deviation) or associated estimates of uncertainty (e.g. confidence intervals)
☐	☒	For null hypothesis testing, the test statistic (e.g. *F*, *t*, *r*) with confidence intervals, effect sizes, degrees of freedom and *P* value noted<br>*Give P values as exact values whenever suitable.*
☒	☐	For Bayesian analysis, information on the choice of priors and Markov chain Monte Carlo settings
☒	☐	For hierarchical and complex designs, identification of the appropriate level for tests and full reporting of outcomes
☒	☐	Estimates of effect sizes (e.g. Cohen's *d*, Pearson's *r*), indicating how they were calculated

Our web collection on statistics for biologists contains articles on many of the points above.

Software and code

Policy information about availability of computer code

Data collection	Zen Blue edition (Carl Zeiss Microscopy) was used for epifluorescence imaging. ImageJ was used to quantify epifluorescent images. Radioactive measurements were analyzed using a liquid scintillation counter. Oxygen consumption data was collected using an XF24 flux analyzer.
Data analysis	Statistical analyses were performed using GraphPad Prism (Graphpad Software v.8.0). LCMS peaks were quantified (area under the curve) using the XCalibur 4.0 (Thermo Scientific) software platform.

For manuscripts utilizing custom algorithms or software that are central to the research but not yet described in published literature, software must be made available to editors/reviewers. We strongly encourage code deposition in a community repository (e.g. GitHub). See the Nature Research guidelines for submitting code & software for further information.

Data

Policy information about availability of data

All manuscripts must include a data availability statement. This statement should provide the following information, where applicable:

- Accession codes, unique identifiers, or web links for publicly available datasets
- A list of figures that have associated raw data
- A description of any restrictions on data availability

Transcriptomic data are made publicly available (EGAS00001003785), other data that support the findings of this study are available in the source data file.

Field-specific reporting

Please select the one below that is the best fit for your research. If you are not sure, read the appropriate sections before making your selection.

[x] Life sciences [ ] Behavioural & social sciences [ ] Ecological, evolutionary & environmental sciences

For a reference copy of the document with all sections, see nature.com/documents/nr-reporting-summary-flat.pdf

Life sciences study design

All studies must disclose on these points even when the disclosure is negative.

Sample size	All data resulted from at least 3 independent differentiations consisting out of at least 3 technical replicates, except the RNA-sequencing experiment data resulted different technical replicates from one biological replicate (one independent differentiation).
Data exclusions	No data exclusion.
Replication	All attempts at replication were successfully confirming the findings presented in this study.
Randomization	As we were specifically comparing different cell types/ states or patient versus controls, randomization is not applicable to the current study.
Blinding	The experiments were not performed with blinding.

Reporting for specific materials, systems and methods

We require information from authors about some types of materials, experimental systems and methods used in many studies. Here, indicate whether each material, system or method listed is relevant to your study. If you are not sure if a list item applies to your research, read the appropriate section before selecting a response.

Materials & experimental systems

n/a	Involved in the study	
[ ]	[x]	Antibodies
[ ]	[x]	Eukaryotic cell lines
[x]	[ ]	Palaeontology
[x]	[ ]	Animals and other organisms
[x]	[ ]	Human research participants
[x]	[ ]	Clinical data

Methods

n/a	Involved in the study	
[x]	[ ]	ChIP-seq
[x]	[ ]	Flow cytometry
[x]	[ ]	MRI-based neuroimaging

Antibodies

Antibodies used	Smi32 (Abcam), Isl1 (Merck Millipore), Tuj1 (Abcam), Chat (Merck Millipore), Synapsin 1 (Merck Millipore)
Validation	Validation data are shown for each antibody by the manufacturers who provide lists of publications. For each antibody, we checked in immunofluorescence analyses that the cellular localisation responded to the reported region of expression.

Eukaryotic cell lines

Policy information about cell lines

Cell line source(s)	Primary human fibroblasts to generate iPSCs were obtained from skin biopsies of ALS patients and controls with the approval of the ethical committee of the University Hospitals Leuven (S50354)
Authentication	The origin of the iPSC lines was confirmed by single nucleotide polymorphism analysis, and presence of the point mutation was validated by sanger sequencing.
Mycoplasma contamination	Cultures were routinely analysed for mycoplasma contamination by PCR.
Commonly misidentified lines (See ICLAC register)	Not applicable.
