# Supplementary material for: Differentiation but not ALS mutations in FUS rewires motor neuron metabolism
Source: Nat Commun. 2019 Sep 12;10:4147. doi: 10.1038/s41467-019-12099-4 (PMC6742665; doi:10.1038/s41467-019-12099-4)
Supplement: Supplementary file 2 — Peer Review [file 41467_2019_12099_MOESM2_ESM.pdf]

Reviewers' comments:

Reviewer #1 (Remarks to the Author):

This paper describes the energy requirements of ips cell lines carrying FUS mutations. The experiments are well performed with appropriate quality control. Notable strengths of the paper include the use of multiple cell lines from multiple individuals, the use of C14 radioactivity to accurately map the energy use and metabolites within the neuronal cell lines, the use of isogenic controls and other controls, and of course the fact that the mutant FUS is not overexpressed (clearly this has led to issues that are rampant in the literature). I do not know of any other group that has leveraged IPS cell lines in this manner, so this is a novel use. The finding that energy metabolism does not alter with FUS mutations is both novel and interesting. I am strongly supportive of negative studies provided that they are well done (as in this instance), as they do provide crucial data points.

I have no major concerns with this manuscript. Minor concerns are as follows:

- To be frank, the manuscript is a bit dense in places, though this is probably context appropriate for this type of manuscript. I found figures 2a and 5a to be very helpful and these should be prominent in the manuscript.
- One issue that comes to mind is whether the motor neurons in a cell dish actually represent the motor neurons in the human body. There is a lack of support cells and this is a rather harsh environment. The authors cannot be faulted for this and they have done an interesting, novel experiment well, but this issue should be mentioned in the discussion, allowing for the possibility that energy metabolism in motor neurons in the human body may be different.
- Not necessarily for inclusion in the manuscript, but I wonder if MRS of humans might be able to show pyruvate or lactate levels.
- As to why only half of the carbons were radioactively labeled (page 6), I wonder if the starting labeled glucose and lactate was really fully labeled. The authors do say on page 5 that it was “uniformly labeled”, but does the company provide any assurances of this?
- The paragraph on mitochondrial morphology seems incongruous. Consider moving to supplementary data.

Reviewer #2 (Remarks to the Author):

In this study, the authors performed a detailed analysis of the energy metabolism in control iPSCs and respective differentiated motor neurons and found that motor neurons preferentially use oxidative metabolism. Then, they performed the same experiments in motor neurons derived from patients with 2 different FUS mutations compared to their respective isogenic controls. Here they found that the FUS mutations did not have any effect on the energy metabolism parameters tested.
While the study was carefully performed the outcome is not surprising considering the authors used very young neurons. The studies where axonal transport defects and hiper-/hypoexcitability were identified used older motor neurons; 7 weeks of differentiation compared to 4 weeks in the current study. Therefore, it would be more informative if the authors can find a time point were the changes occur.
As for the first part of the study, and even though I agree that this is a thorough characterization of the energy metabolism in iPSCs and motor neurons, the finding that iPSCs switch from a more glycolytic metabolism to oxidative phosphorylation in neurons is not a new concept. The authors acknowledge this as well in the discussion section. Overall the questions the authors attempted to tackle are important, but the study lacks novelty.

Response to the referees

Reviewer #1

This paper describes the energy requirements of iPS cell lines carrying FUS mutations. The experiments are well performed with appropriate quality control. Notable strengths of the paper include the use of multiple cell lines from multiple individuals, the use of C14 radioactivity to accurately map the energy use and metabolites within the neuronal cell lines, the use of isogenic controls and other controls, and of course the fact that the mutant FUS is not overexpressed (clearly this has led to issues that are rampant in the literature). I do not know of any other group that has leveraged iPS cell lines in this manner, so this is a novel use. The finding that energy metabolism does not alter with FUS mutations is both novel and interesting. I am strongly supportive of negative studies provided that they are well done (as in this instance), as they do provide crucial data points. I have no major concerns with this manuscript.

We would like to thank the reviewer for this positive evaluation of our work.

Minor concerns:

1. *To be frank, the manuscript is a bit dense in places, though this is probably context appropriate for this type of manuscript. I found figures 2a and 5a to be very helpful and these should be prominent in the manuscript.*

We thank the referee for this comment and improved the readability of our manuscript by adding several explanatory statements into the revised version. We also removed the data describing the composite nature of ATP as these results did not contribute to our main story (which was to explain energy metabolic rewiring upon motor neuron differentiation) and as such had a negative effect on the readability of our paper.

2. *One issue that comes to mind is whether the motor neurons in a cell dish actually represent the motor neurons in the human body. There is a lack of support cells and this is a rather harsh environment. The authors cannot be faulted for this and they have done an interesting, novel experiment well, but this issue should be mentioned in the discussion, allowing for the possibility that energy metabolism in motor neurons in the human body may be different.*

We fully agree with the referee and we clarified this further in the discussion as highlighted in our revised manuscript:

"Indeed, ALS is a complex disease in which various cell types interact to induce MN degeneration. For example, oligodendrocytes, which are vital to metabolically support MNs, are implicated in ALS pathogenesis. (…) Results from patient-derived MNs should therefore be interpreted with the limitations of the current model in mind. Future studies using more complex patient-derived culture systems, which better mimic the MN micro-environment by for example including glial cells, could be required to address these outstanding questions."

3. *Not necessarily for inclusion in the manuscript, but I wonder if MRS of humans might be able to show pyruvate or lactate levels.*

We agree with the referee that using magnetic resonance spectroscopy (MRS) is a potent technique for the non-invasive *in vivo* investigation of tissue chemistry and central nervous system metabolism.

However, while brain lactate can be measured using MRS[1-3], we could not find any paper measuring pyruvate concentrations in the central nervous system using MRS. This is most likely due to the low signal/noise ratio inherent to MRS technology making the detection of less abundant metabolites challenging[4]. While assessing metabolite (mainly N-acetyl aspartate) concentrations by proton MRS has been used to follow-up motor neuron degeneration in ALS patients, we are unaware of any MRS study measuring CNS lactate levels in ALS patients[5,6].

4. *As to why only half of the carbons were radioactively labeled (page 6), I wonder if the starting labeled glucose and lactate was really fully labeled. The authors do say on page 5 that it was "uniformly labeled", but does the company provide any assurances of this?*

While Cambridge Isotope Laboratories assures uniform labeling by providing a certificate of analysis for every lot number, this is indeed a justified comment. We assured uniform labeling by performing different quality control experiments.

First, we validated whether glucose and lactate were indeed absent from our glucose-free media. We analyzed glucose and lactate abundance in four different media samples by LCMS. One sample contained a 1:1 mixture of conventional DMEM/F12 and Neurobasal, while the three other samples were a 1:1 mixture of glucose-free DMEM/F12 and Neurobasal to which we added 2 mM lactate with either 0, 5 or 10 mM glucose. We confirmed that our glucose free medium indeed did not contain glucose nor lactate (Revision Fig. 1a).

Second, we ruled out the possibility of ^{12}C contamination of our uniformly labelled glucose and lactate isotope tracers. To do this, we measured the fractional contribution (Revision Fig. 1b) of ^{13}C to glucose and lactate and the relative abundance of glucose and lactate isotopologues (Revision Fig. 1c) in media samples containing 5 mM ^{12}C or $^{13}C_6$- glucose supplemented with either 2 mM ^{12}C or $^{13}C_3$- lactate. These results clearly confirm that both the $^{13}C_6$- glucose as well as the $^{13}C_3$- lactate were indeed uniformly labeled and media was not contaminated with ^{12}C glucose or with ^{12}C lactate.

As we agree with the referee that this is indeed important information, we stated in the LCMS method section that we performed quality control experiments confirming that our isotope tracers were uniformly labelled.

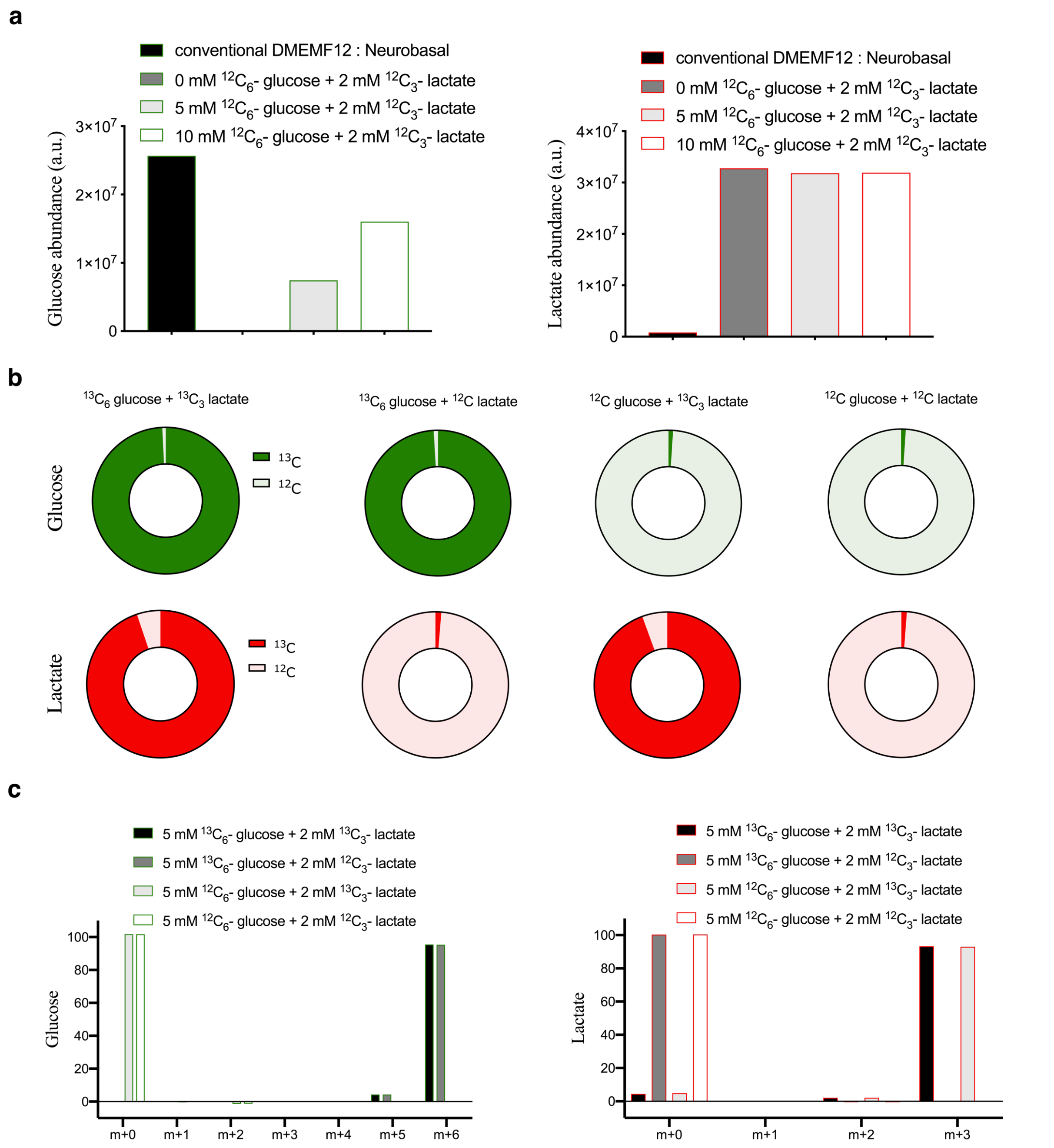

Revision Fig. 1. Quality control of glucose free media and ^{13}C isotope tracers. a, Abundance of glucose and lactate in culture media to which different concentrations of glucose and lactate were added. **b,** Fractional contribution of $^{13}C_6$ glucose (dark green) and $^{13}C_3$ lactate (dark red) to glucose and lactate in culture media containing either 5 mM $^{13}C_6$ or 5 mM ^{12}C glucose with 2 mM $^{13}C_3$ or 2 mM ^{12}C lactate. **c,** Relative abundance of each isotopologue for glucose and lactate in culture media containing 5mM $^{13}C_6$ or ^{12}C glucose with 2mM $^{13}C_3$ or ^{12}C lactate.

5. *The paragraph on mitochondrial morphology seems incongruous. Consider moving to supplementary data.*

We believe that the data assessing the direct effect of FUS-ALS causing mutations on mitochondrial morphology in human FUS motor neurons are important. First, aberrant mitochondrial morphology has been observed in different ALS models and in *post mortem* patient material[7-9]. Second, recent studies reported disturbed mitochondrial morphology upon overexpression of mutant or wildtype FUS, suggesting that mutant FUS might abrogate normal mitochondrial morphology in motor neurons[10,11]. Third, the effect of a FUS-ALS mutation on mitochondrial morphology has never been assessed in human motor neuron using isogenic controls. Therefore, we prefer to keep our observation in the main text.

However, we agree with the referee that the relevance of our TEM data should be introduced and integrated better and we have done this by including the following statement:

“While we previously reported that mitochondrial morphology was unaltered in FUS-mutant lines compared to healthy MNs[12], recent data showed that FUS overexpression impairs mitochondrial structure[11,13]. Moreover, mitochondrial dysfunction is a clinical hallmark of ALS (for a review see:[14]). Therefore, we assessed the direct effect of FUS-ALS causing point mutations on mitochondrial morphology using transmission electron microscopy focusing in FUS lines and isogenic controls.”

Reviewer #2:

In this study, the authors performed a detailed analysis of the energy metabolism in control iPSCs and respective differentiated motor neurons and found that motor neurons preferentially use oxidative metabolism. Then, they performed the same experiments in motor neurons derived from patients with 2 different FUS mutations compared to their respective isogenic controls. Here they found that the FUS mutations did not have any effect on the energy metabolism parameters tested.

While the study was carefully performed the outcome is not surprising considering the authors used very young neurons. The studies where axonal transport defects and hyper-/hypoexcitability were identified used older motor neurons; 7 weeks of differentiation compared to 4 weeks in the current study. Therefore, it would be more informative if the authors can find a time point were the changes occur.

The reviewer indicated that there is a difference of 3 weeks between our current experiments and the time at which we observe different disease related phenotypes[12]. This is not completely true, and we believe that this confusion might have arisen from the use of "weeks post-plating" and "days of differentiation" interchangeably.

In the current experiments all motor neurons were between 28 and 32 days after initiation of differentiation. The timing of the phenotypes we observed in our previous paper is as follows:

1. Mislocalization of FUS protein was already observed in motor neurons during the fourth week after starting differentiation (day 21–28), but also in patient fibroblasts and iPSCs. This phenotype therefore matches the timing of the metabolic experiments in our current submission.
2. Axonal transport defects were present from the third week post-plating. As plating occurs at day 10, three weeks post plating means that axonal transport defects were present as from day 31 of differentiation onwards. Hence, the timepoint at which we observe axonal transport defects also corresponds to the timepoint of the experiments presented in our initial submission.
3. Electrophysiological experiments were carried out in the seventh week after starting differentiation (from day 43 of differentiation)[12]. This is indeed beyond the timepoint at which we performed metabolic experiments.

We therefore agree with the reviewer that while our current study matches the time points at which we observe axonal transport defect and FUS mislocalization, they are indeed performed prior to the time at which we observed hypoexcitability. Also, as the axonal transport defect we observe is progressive beyond the timepoint at which we performed our metabolic studies, we agree that it would have been valuable to assess whether or not metabolic defects arise at a later time point.

To address this outstanding issue, we performed all our metabolic studies (transmission electron microscopy, oxygen consumption measurements, $^{13}C_6$- glucose and $^{13}C_3$- lactate fate mapping, and radiolabeling studies assessing: glucose uptake, glycolytic flux, glucose oxidation, lactate oxidation, glutamine oxidation, fatty acid oxidation) during the seventh week after starting differentiation (day 42–46). We chose to repeat these experiments using motor

neurons derived from the patient carrying the P525L mutation as this patient was not only clinically the most severely affected but also showed more pronounced FUS mislocalization.

None of these experiments showed a difference between the P525L patient line and its isogenic control. We wish to thank the reviewer for this important suggestion. We mention this in the main paper at page 9 and added the data in an additional supplementary figure (Supplementary Fig. 4) as it clearly strengthens our conclusions.

As for the first part of the study, and even though I agree that this is a thorough characterization of the energy metabolism in iPSCs and motor neurons, the finding that iPSCs switch from a more glycolytic metabolism to oxidative phosphorylation in neurons is not a new concept. The authors acknowledge this as well in the discussion section. Overall the questions the authors attempted to tackle are important, but the study lacks novelty.

We agree that a switch from a glycolytic to a more oxidative metabolic phenotype upon differentiation has been observed in other cell types, as we mentioned in the manuscript:

“Energy metabolism is considered to be an important regulator during the acquisition of stemness[15,16], as well as during differentiation into specialized cell types[17,18]. While a shift from a glycolytic to an oxidative metabolic state during the differentiation of primary mouse or iPS cells to cortical neurons was recently shown, evidence supporting the presence of a pro-oxidative shift upon MN differentiation is limited.”

However, ALS is characterized by the selective degeneration of motor neurons and metabolic dysregulation[19]. Nevertheless, metabolic characteristics of neurons are almost exclusively studied in cortical neurons[20,21]. The **energy metabolism of human motor neurons has never been functionally characterized**. Since the primary aim of our work was to study if FUS-ALS causing point mutations affect energy metabolism of human motor neurons, we believe it was crucial to first gain insight in the energy metabolism of human motor neurons specifically. We are convinced that this is a vital step which should not be overlooked because a cell type specific characterization of metabolic switching upon differentiation is likely to be essential to fully understand the metabolic phenotype of a particular cell type, both in health and disease.

Additional novelty arises as we studied metabolic rewiring upon motor neuron differentiation in an **energy substrate specific** manner. To the best of our knowledge, this has never been done before in motor neurons, nor any other neuronal subtype. By doing so, we came to the novel findings that, first, the pro-oxidative switch upon motor neuron differentiation is **mainly fuelled by an increase in lactate oxidation** and, second, that **pathways to import pyruvate into the TCA cycle rewire differently depending on the energy substrate**.

We believe that our data add novel information which is needed to provide a basis to interpret our and future findings regarding motor neuron metabolism in ALS. To clarify all this to the reader we rephrased different statements as highlighted in our revised version on pages: 2, 4, 6, and 10.

References:

1 Chu, W. J. *et al.* Magnetic resonance spectroscopy imaging of lactate in patients with bipolar disorder. *Psychiatry Res* **213**, 230-234, doi:10.1016/j.pscychresns.2013.03.004 (2013).

2 Maddock, R. J., Casazza, G. A., Buonocore, M. H. & Tanase, C. Vigorous exercise increases brain lactate and Glx (glutamate+glutamine): a dynamic 1H-MRS study. *Neuroimage* **57**, 1324-1330, doi:10.1016/j.neuroimage.2011.05.048 (2011).

3 Prichard, J. *et al.* Lactate rise detected by 1H NMR in human visual cortex during physiologic stimulation. *Proc Natl Acad Sci U S A* **88**, 5829-5831, doi:10.1073/pnas.88.13.5829 (1991).

4 Osorio-Garcia, M. I., Sima, D. M., Nielsen, F. U., Himmelreich, U. & Van Huffel, S. Quantification of magnetic resonance spectroscopy signals with lineshape estimation. *J Chemometr* **25**, 183-192, doi:10.1002/cem.1353 (2011).

5 Pohl, C. *et al.* Proton magnetic resonance spectroscopy of the motor cortex in 70 patients with amyotrophic lateral sclerosis. *Arch Neurol* **58**, 729-735 (2001).

6 Kalra, S. Magnetic Resonance Spectroscopy in ALS. *Front Neurol* **10**, 482, doi:10.3389/fneur.2019.00482 (2019).

7 Bowling, A. C., Schulz, J. B., Brown, R. H., Jr. & Beal, M. F. Superoxide dismutase activity, oxidative damage, and mitochondrial energy metabolism in familial and sporadic amyotrophic lateral sclerosis. *J Neurochem* **61**, 2322-2325 (1993).

8 Dupuis, L. *et al.* Mitochondria in amyotrophic lateral sclerosis: a trigger and a target. *Neurodegener Dis* **1**, 245-254, doi:10.1159/000085063 (2004).

9 Sasaki, S., Horie, Y. & Iwata, M. Mitochondrial alterations in dorsal root ganglion cells in sporadic amyotrophic lateral sclerosis. *Acta Neuropathol* **114**, 633-639, doi:10.1007/s00401-007-0299-1 (2007).

10 So, E. *et al.* Mitochondrial abnormalities and disruption of the neuromuscular junction precede the clinical phenotype and motor neuron loss in hFUSWT transgenic mice. *Hum Mol Genet*, doi:10.1093/hmg/ddx415 (2017).

11 Deng, J. *et al.* FUS interacts with ATP synthase beta subunit and induces mitochondrial unfolded protein response in cellular and animal models. *Proc Natl Acad Sci U S A* **115**, E9678-E9686, doi:10.1073/pnas.1806655115 (2018).

12 Guo, W. *et al.* HDAC6 inhibition reverses axonal transport defects in motor neurons derived from FUS-ALS patients. *Nat Commun* **8**, 861, doi:10.1038/s41467-017-00911-y (2017).

13 Mitchell, J. C. *et al.* Overexpression of human wild-type FUS causes progressive motor neuron degeneration in an age- and dose-dependent fashion. *Acta Neuropathol* **125**, 273-288, doi:10.1007/s00401-012-1043-z (2013).

14 Smith, E. F., Shaw, P. J. & De Vos, K. J. The role of mitochondria in amyotrophic lateral sclerosis. *Neurosci Lett*, doi:10.1016/j.neulet.2017.06.052 (2017).

15 Panopoulos, A. D. *et al.* The metabolome of induced pluripotent stem cells reveals metabolic changes occurring in somatic cell reprogramming. *Cell Res* **22**, 168-177, doi:10.1038/cr.2011.177 (2012).

16 Folmes, C. D. *et al.* Somatic oxidative bioenergetics transitions into pluripotency-dependent glycolysis to facilitate nuclear reprogramming. *Cell Metab* **14**, 264-271, doi:10.1016/j.cmet.2011.06.011 (2011).

17 Varum, S. *et al.* Energy metabolism in human pluripotent stem cells and their differentiated counterparts. *PLoS One* **6**, e20914, doi:10.1371/journal.pone.0020914 (2011).
18 Zhang, J. *et al.* UCP2 regulates energy metabolism and differentiation potential of human pluripotent stem cells. *EMBO J* **35**, 899, doi:10.15252/embj.201694054 (2016).
19 Vandoorne, T., De Bock, K. & Van Den Bosch, L. Energy metabolism in ALS: an underappreciated opportunity? *Acta Neuropathol* **135**, 489-509, doi:10.1007/s00401-018-1835-x (2018).
20 Zheng, X. *et al.* Metabolic reprogramming during neuronal differentiation from aerobic glycolysis to neuronal oxidative phosphorylation. *Elife* **5**, doi:10.7554/eLife.13374 (2016).
21 Agostini, M. *et al.* Metabolic reprogramming during neuronal differentiation. *Cell Death Differ* **23**, 1502-1514, doi:10.1038/cdd.2016.36 (2016).

REVIEWERS' COMMENTS:

Reviewer #1 (Remarks to the Author):

The authors have adequately addressed the comments of the reviewers and the manuscript is improved. In particular, performing the experiments at a later time point and the QC measures are additional data points that add credence to the authors' conclusions. This is a well-presented and important data point for the community that moves the energy metabolism debate beyond the clinical realm and into a testable, experimental setting.

Reviewer #2 (Remarks to the Author):

I am satisfied with the authors responses and have no further comments or questions.

Response to the referees

Reviewer #1 (Remarks to the Author):

The authors have adequately addressed the comments of the reviewers and the manuscript is improved. In particular, performing the experiments at a later time point and the QC measures are additional data points that add credence to the authors' conclusions. This is a well-presented and important data point for the community that moves the energy metabolism debate beyond the clinical realm and into a testable, experimental setting.

Reviewer #2 (Remarks to the Author):

I am satisfied with the authors responses and have no further comments or questions.

We would like to thank the reviewers for this positive evaluation of our work and for their thorough evaluation and constructive remarks throughout the entire reviewing process.
